# Semi-Quantitative Targeted Gas Chromatography-Mass Spectrometry Profiling Supports a Late Side-Chain Reductase Cycloartenol-to-Cholesterol Biosynthesis Pathway in Brown Algae

**DOI:** 10.3389/fpls.2021.648426

**Published:** 2021-04-27

**Authors:** Jean Girard, Goulven Lanneau, Ludovic Delage, Cédric Leroux, Arnaud Belcour, Jeanne Got, Jonas Collén, Catherine Boyen, Anne Siegel, Simon M. Dittami, Catherine Leblanc, Gabriel V. Markov

**Affiliations:** ^1^CNRS, Integrative Biology of Marine Models (LBI2M, UMR8227), Station Biologique de Roscoff (SBR), Sorbonne Université, Roscoff, France; ^2^CNRS, Plateforme Corsaire-METABOMER (FR2424), Station Biologique de Roscoff, Sorbonne Université, Roscoff, France; ^3^Univ Rennes, Inria, CNRS, IRISA, Equipe Dyliss, Rennes, France

**Keywords:** sterol, brown alga, gas chromatography-mass spectrometry, biosynthesis pathway evolution, side-chain reduction

## Abstract

Sterols are biologically important molecules that serve as membrane fluidity regulators and precursors of signaling molecules, either endogenous or involved in biotic interactions. There is currently no model of their biosynthesis pathways in brown algae. Here, we benefit from the availability of genome data and gas chromatography-mass spectrometry (GC-MS) sterol profiling using a database of internal standards to build such a model. We expand the set of identified sterols in 11 species of red, brown, and green macroalgae and integrate these new data with genomic data. Our analyses suggest that some metabolic reactions may be conserved despite the loss of canonical eukaryotic enzymes, like the sterol side-chain reductase (SSR). Our findings are consistent with the principle of metabolic pathway drift through enzymatic replacement and show that cholesterol synthesis from cycloartenol may be a widespread but variable pathway among chlorophyllian eukaryotes. Among the factors contributing to this variability, one could be the recruitment of cholesterol biosynthetic intermediates to make signaling molecules, such as the mozukulins. These compounds were found in some brown algae belonging to Ectocarpales, and we here provide a first mozukulin biosynthetic model. Our results demonstrate that integrative approaches can already be used to infer experimentally testable models, which will be useful to further investigate the biological roles of those newly identified algal pathways.

## Introduction

Sterols are biologically important molecules in eukaryotes, which function as membrane fluidity regulators and precursors of signaling molecules, either endogenous or involved in biotic interactions. The associated biosynthetic pathways are good models to study metabolic pathway evolution because the enzymes involved are well-known across eukaryotes and relatively conserved despite some variation (Desmond and Gribaldo, 2009). Recently, several studies have enabled to better understand the history of this pathway in land plants (Sonawane et al., 2016), diatoms (Gallo et al., 2020), or red algae (Belcour et al., 2020). However, a general model for sterol biosynthesis in brown algae – a vast diversified and independent eukaryotic lineage (Bringloe et al., 2020) – is still lacking.

The main brown algal sterol is fucosterol, but C28 and C27 sterols have also been reported, for example, in the model brown alga *Ectocarpus siliculosus* (Mikami et al., 2018). Among C27 derivatives, specialized metabolites have also been identified, such as a molecule bearing structural similarity to a brassinosteroid biosynthesis precursors in the fucale *Cytoseira myrica* (Hamdy et al., 2009), or the mozukulins from another ectocarpale species, the mozuku *Cladosiphon okamuranus* (Cheng et al., 2016). Some of these specialized metabolites could be important signaling or defense molecules, but so far there is little knowledge about their potential biological roles (Markov et al., 2018).

In order to build sterol biosynthesis pathway models that take into account data on metabolite distribution, we carried out semi-quantitative gas chromatography-mass spectrometry (GC-MS) profiling of sterols present in the two brown algal orders, the Ectocarpales and the Laminariales, for which genome-scale metabolic networks have been recently reconstructed (Aite et al., 2018; Nègre et al., 2019; Dittami et al., 2020). Specifically, we explored the sterol profiles of the two *Ectocarpus* species for which genome sequences are available, *E. siliculosus* and *Ectocarpus subulatus*, as well as two local species from Roscoff: *Ectocarpus fasciculatus* and *Ectocarpus crouanorium*, and one additional member from the Ectocarpales order: *Pylaiella littoralis*. We also explored the sterol profiles of two kelp species (Laminariales) from Roscoff: *Saccharina latissima* and *Laminaria digitata*. Furthermore, we analyzed these profiles in three red algae (*Chondrus crispus*, *Mastocarpus stellatus*, and *Palmaria palmata*), and one green alga (*Ulva* sp.), as a basis for comparison and to further investigate the intriguing issue of undetectable cycloartenol that we noticed previously in *C. crispus* (Belcour et al., 2020).

## Materials and Methods

### Sampling of Algae

Macroalgal samples from *L. digitata*, *Ulva* sp., *C. crispus*, and *M. stellatus* were collected at low tides on the shore at Roscoff, France, in front of the Station Biologique (48°43′38″ N; 3°59′04″ W) for *Ulva* sp., *C. crispus*, and *M. stellatus*, and at the Bloscon site (48°43“31”N; 3°58“8”W) for *L. digitata*. Samples from *E. fasciculatus* and *E. crouaniorum* came from frozen samples collected at Roscoff (Perharidy site, 48.73° N, 4.00° W) in 2009. Samples of *S. latissima*, *E. subulatus* Ec371 (CCAP accession 1310), *E. siliculosus* Ec32 (CCAP accession 1310/04), and *P. palmata* came from cultures and were maintained in 10 L Nalgene flasks in a culture room at 14°C using filtered and autoclave seawater enriched with Provasoli nutrients (Bold and Wynne, 1978) and aerated with 0.22 μm-filtered compressed air to avoid CO_2_ depletion. The cultures of *S. latissima* were started from freshly released spores of mature sporophytes collected at Perharidy as described previously (Bernard et al., 2018). Photosynthetically active radiation was provided by Philips daylight fluorescence tubes at a photon flux density of 40 μmol.m^−2^.s^−1^ for 14 h.d^−1^. The algal samples were freeze-dried, ground to powder and stored at −80°C up to chemical extraction.

### Standards and Reagents

Cholesterol, stigmasterol, β-sitosterol, 7-dehydrocholesterol, lathosterol (5α-cholest-7-en-3β-ol), squalene, campesterol, brassicasterol, desmosterol, lanosterol, fucosterol, cycloartenol, and 5α-cholestane (internal standard) were acquired from Sigma-Aldrich (Saint-Quentin-Fallavier, France), cycloartanol and cycloeucalenol from Chemfaces (Wuhan, China), and zymosterol from Avanti Polar Lipids (Alabaster, United States). The C7-C40 Saturated Alkanes Standards were acquired from Supelco (Bellefonte, United States). Reagents used for extraction, saponification, and derivation steps were n-hexane, ethyl acetate, acetonitrile, methanol (Carlo ERBA Reagents, Val de Reuil, France), (trimethylsilyl)diazomethane, toluene (Sigma-Aldrich) and N,O-bis(trimethylsilyl)trifluoroacetamide with trimethylcholorosilane [BSTFA:TMCS (99:1); Supelco].

### Standard Preparation

Stock solutions of cholesterol, stigmasterol, β-sitosterol, 7-dehydrocholesterol, lathosterol (5α-cholest-7-en-3β-ol), squalene, campesterol, brassicasterol, desmosterol, lanosterol, fucosterol, cycloartenol, and 5α-cholestane were prepared in hexane at a concentration of 5 mg.ml^−1^. Working solutions were made at a concentration of 1 mg.ml^−1^, in hexane, by diluting stock solutions. The C7-C40 Saturated Alkanes Standard stock had a concentration of 1 mg.ml^−1^ and a working solution was prepared at a concentration of 0.1 mg.ml^−1^. All solutions were stored at −20°C. The 24-alkylsterols from algae are often epimers from their land plant counterparts that can be distinguished by NMR (Rubinstein et al., 1976; Nes, 2011), so here campesterol, brassicasterol, sitosterol, and stigmasterol were used as proxies for both epimers of, respectively, 24-methylcholesterol, 24-methylcholest-22-enol, 24-ethylcholesterol and 24-ethylcholest-22-enol.

### Calibration of Standard Quantification Curves

To avoid biases linked to the variation of the behavior of individual sterols during the ionization step, a quantification method specific to the available standards was developed, according to the recommendations of the community (Khoury et al., 2018). We choosed to use a method with a correction on average standard curves between mix standards, which contained all sterols of the global mix standards, but divided in two new mixes with time retention well distinct, because this appears to give the best accurate relative quantification (details are given in Supplementary Material, Supplementary Figures S1–S5 and Supplementary Tables S1–S5).

### Sample Preparation

Algal samples (60 mg) were extracted with 2 ml ethyl acetate by continuous agitation for 1 h at 4°C. After 10 min of centrifugation at 4,000 rpm, the solvent was removed, the extracts were saponified in 3 ml of methanolic potassium hydroxide solution (1 M) during 1 h of incubation at 90°C. The saponification reaction was stopped by plunging samples into an ice bath for 30 min minimum. The unsaponifiable fraction was extracted with 2 ml of hexane and 1.2 ml of water and centrifuged at 2,000 rpm for 5 min. The upper phase was collected, dried under N_2_, and resuspended with 120 μl of (trimethylsilyl)diazomethane, 50 μl of methanol:toluene [2:1 (v/v)] and 5 μl of 5α-cholestane (1 mg.ml^−1^) as internal standard. The mixture was vortexed for 30 s and then heated to 37°C for 30 min. After a second evaporation under N_2_, 50 μl of acetonitrile and 50 μl of BSTFA:TMCS (99:1) were added to the dry residue, vortexed for 30 s and heated to 60°C for 30 min. After final evaporation under N_2_, the extract was resuspended in 100 μl of hexane, transferred into a sample vial and stored at −80°C until the GC-MS analysis.

### Gas Chromatography-Mass Spectrometry Analysis

The sterols were analyzed on a 7890 Agilent Technologies gas chromatography coupled with a 5975C Agilent Technologies mass spectrometer (GC-MS). A HP-5MS capillary GC column (30 m × 0.25 mm × 0.25 μm) from J&W Scientific (CA, United States) was used for separation and UHP helium was used as carrier gas at a flow rate to 1 ml.min^−1^. The temperature of the injector was 280°C and the detector temperature was 315°C. After injection, the oven temperature was kept at 60°C for 1 min. The temperature was increased from 60 to 100°C at a rate of 25°C.min^−1^, then to 250°C at a rate of 15°C.min^−1^, then to 315°C at a rate of 3°C.min^−1^ and then held at 315°C for 2 min, resulting in a total run time of 37 min. Electronic impact mass spectra were measured at 70 eV and an ionization temperature of 250°C. The mass spectra were scanned from m/z 50 to m/z 500. Peaks were identified based on the comparisons with the retention times and the mass spectra (Supplementary Table S1).

### Searches for Orthologous Genes

Similarity searches for orthologous genes were performed using BLAST either against protein sequences, when available, or directly on translated nucleotidic sequences on the sequence read archive (SRA) or transcriptome shotgun assemblies (TSA). Project accession numbers for SRA and TSA are indicated in Supplementary Table S6.

### *Ab-initio* Inference of Biosynthesis Pathways

*Ab-initio* inference of biosynthesis pathway was performed using the Pathmodel program (Belcour et al., 2020). The encoding source files for the mozukulins pathway and the brown sterol biosynthetic pathways were added in a new version of Pathmodel (0.2.0). See full links in the data availability statement.

### Ancestral Character Mapping

Mitochondrial *cox3* nucleotidic sequences from GenBank (Accession numbers in Supplementary Table S7) were aligned using Clustal Omega (Sievers and Higgins, 2014) as implemented in Seaview version 4.5.4 (Gouy et al., 2010). Phylogenetic trees were built using PHYML 3.1 (Guindon and Gascuel, 2003) using the GTR with a gamma law and estimation of the proportion of invariable sites. Ancestral character reconstruction and stochastic mapping (Huelsenbeck et al., 2003) were performed under R version 3.2.2 (R Core Team, 2015) using the make.simmap function as implemented in the phytools package version 0.5.0 (Revell, 2012). Character evolution was inferred using a model of symmetrical transition rates between the character states (SYM). About 1,000 character histories were sampled to allow the incorporation of the uncertainty associated with the transition between different states. Inferred state frequencies for ancestral nodes were plotted using the describe.simmap function. See the link for commands and sources files in the data availability statement.

### Update of Genome-Scale Metabolic Networks

Update of the genome-scale metabolic networks is available at genouest.org for four brown algal species (*E. siliculosus*, *E. subulatus*, *C. okamuranus*, and *Saccharina japonica*) was performed using the curation function of AuReMe (Aite et al., 2018). The added reactions are also available as Supplementary Material (Supplementary Tables S8–S11).

## Results

### Targeted GC-MS Profiling and Quantification of Sterols in Seven Brown Algae and Four Red and Green Algae

**Figure 1:**
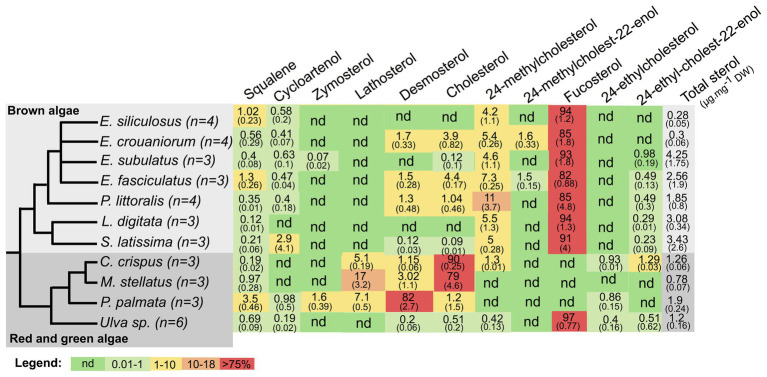
Relative abundances of squalene and sterols detected in algal samples measured by semi-quantitative gas chromatography-mass spectrometry (GC-MS) profiling. Relative abundance values are expressed as a percentage of total sterol content, mean of multiple replicates, with SD in brackets. “nd” means not detectable (<0.01%). The tree represents the consensus view of the species phylogeny, with number of biological replicates indicated for each species. Last column (in gray) indicates the abundance of sterols relative to dry weight (DW).

We identified and quantified 12 sterols plus their common biosynthetic precursor squalene. The total quantity of sterols varied between 0.28 and 4.25 μg.mg^−1^ dry weight (DW) across samples (Figure 1). Among them, seven sterols were found in at least some brown algae, fucosterol being the most abundant in brown and green algae, whereas cholesterol or desmosterol were the most abundant sterols in red algae (Figure 1). In order to get a more synthetic overview of the variation of sterol profiles in a phylogenetic context, we plotted the sterol profiles on a cladogram showing the phylogenetic relationships between the investigated species (Figure 1).

Among the remaining sterols, cycloartenol was found in all algae except *C. crispus*, as previously noticed (Belcour et al., 2020), and *L. digitata*, where we could also interpret this absence as it being below the detection limit. Indeed, its precursor, squalene, is also found in the lowest concentration in *L. digitata* compared to the other examined brown algae. 24-methylcholesterol also turned out to be an important component of brown algal sterol profiles, representing between 4.2 and 11% of total sterols, whereas 24-ethylcholest-22-enol, 24-methylcholest-22-enol, desmosterol, and cholesterol were not always detectable and showed variable patterns. 24-ethylcholest-22-enol was always a rare sterol (between 0 and 0.98% in all brown algae), whereas cholesterol ranged from 0 to 4.4%. Among the other investigated sterols, lanosterol was never found, whereas 24-ethylcholesterol and lathosterol were found only in the three red algal species, and zymosterol was found only in *P. palmata* and in *E. subulatus*. The fact that we did not detect ergosterol in any of the analyzed algae was particularly striking because it was recently reported to be present in *E. siliculosus*, using a different separation technique, HPLC coupled to fluorescence detection (Mikami et al., 2018). Therefore, we checked using a spiked extract that we were technically able to identify the standard in an extract from *E. siliculosus* tissue (Supplementary Figure S1). We also tentatively identified small amounts of coprostanol, a degradation product of cholesterol, in *P. littoralis*, but did not include it in the table because we do not have the corresponding analytical standard. Altogether, our analysis indicates that some closely related species share similar features that are linked to a common evolutionary history. For example, the three red algal species synthesize mainly sterols with 27 carbons, which correlate with the loss of the canonical sterol methyltransferases, transferring additional methyl groups in other lineages (Belcour et al., 2020; Gallo et al., 2020). However, *C. crispus* and *M. stellatus*, which belong to the Gigartinales, produce mainly cholesterol, whereas *P. palmata* belonging to the Palmariales produces mainly desmosterol. For brown algae, the variation across the four species of *Ectocarpus* indicates that, even among close relatives, there can also be important variations both in terms of total sterol content, equivalent to the variation observed across the three phyla, and also in terms of relative sterol abundance. In that case, as we did not specifically control for developmental stage or physiological state among samples, those parameters can contribute as much as phylogenetic history to the observed variation.

### A General Model for Sterol Biosynthesis in Brown Algae

**Figure 2:**
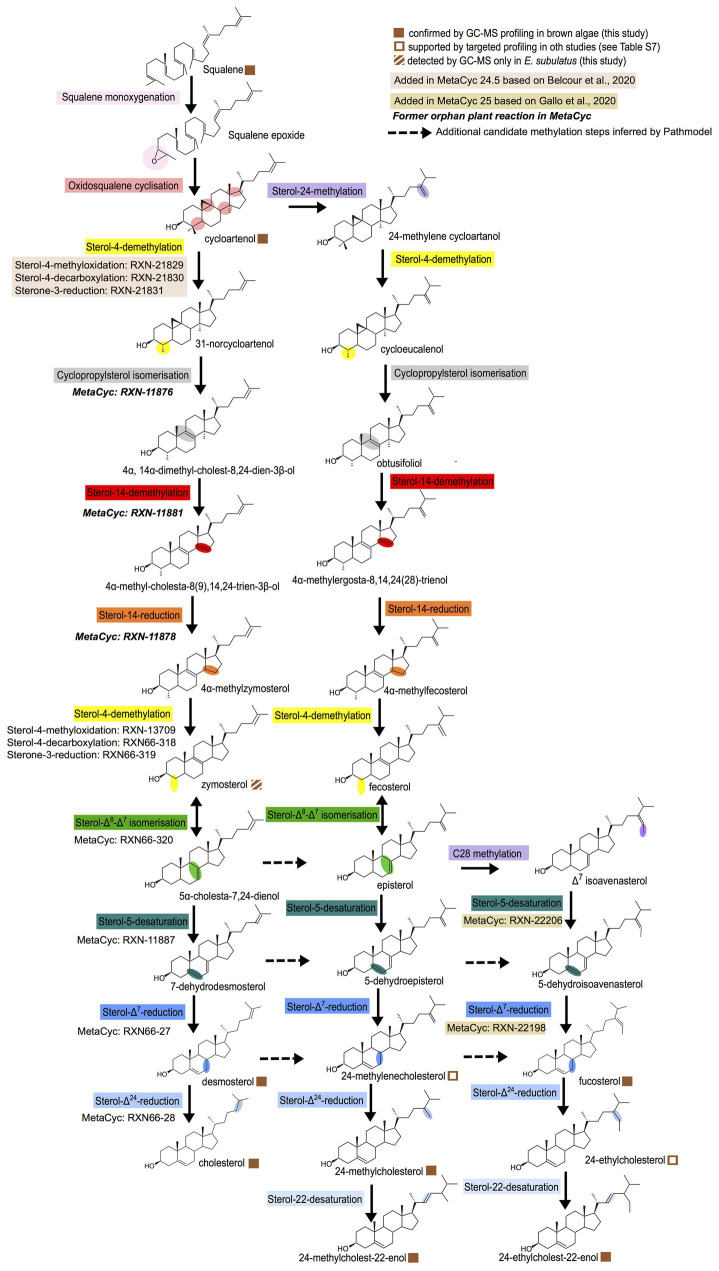
General model for sterol biosynthesis in brown algae. The model was generated with Pathmodel, using the end metabolites isolated by GC-MS as targets and using known reactions. The colors indicate similar molecular transformations occurring on different substrates in the various pathway branches.

In order to build a general biosynthesis model for the brown algal sterols confirmed by GC-MS profiling, we used the previously developed Pathmodel program (Belcour et al., 2020) to infer pathways based on the data about the presence and absence of sterols in brown alga and the related biochemical reactions registered in the MetaCyc database (Caspi et al., 2020). This model shows a mixture of features that are either conserved in other lineages or specific to brown algae (Figure 2).

The biosynthesis pathway from cycloartenol to cholesterol is similar to one of the two alternative pathways potentially present in the red alga *C. crispus* (Belcour et al., 2020), and identical to the biosynthesis pathway proposed independently for two diatoms (Gallo et al., 2020). The difference in both pathways is that, in brown algae and diatoms, the second sterol-4-demethylation occurs before the sterol-Δ^8^-Δ^7^-isomerization, to accommodate the presence of zymosterol that we observed in at least one species (Figure 2), whereas in the red alga, the isomerization was inferred to occur before the demethylation, in line with the lack of zymosterol detection in this species (Belcour et al., 2020). Interestingly, during the incorporation of the *C. crispus* model in the MetaCyc database by manual curation, we noticed that not all reactions needed to be added. Some (in *bold italics* on Figure 2) were already present in the database, as orphan reactions not connected to a pathway. Their presence was established using enzymatic assays on sterol bioconversion performed with cellular extracts (Paik et al., 1984) or using biochemical characterization of recombinant plant enzymes in yeast (Grebenok et al., 1998; Lovato et al., 2000; Rahier et al., 2009). This therefore suggests that a cycloartenol-to-cholesterol pathway with late sterol-Δ^24^-reduction may be present as a whole in plants that synthesize cholesterol, additional to the cycloartenol-to-cholesterol pathway with early sterol-Δ^24^-reduction that has already been proposed for solanaceae (Sonawane et al., 2016).

The biosynthetic pathway from cycloartenol to 24-methylcholest-22-enol shares many steps with the classical land plant biosynthesis pathway from cycloartenol to campesterol (Desmond and Gribaldo, 2009), the main difference being that as for the pathway from cycloartenol to cholesterol, the second sterol-4-demethylation is inferred here to occur before the sterol-Δ^8^-Δ^7^-isomerization. The pathway leading to fucosterol may end up in a diatom-like way with a sterol-5-desaturation and a sterol-Δ^7^-reduction following a sterol-28-methylation leading to different epimers than in land plants (Gallo et al., 2020). However, since the main biochemical requirement for sterol side chain alkylation at carbons 24 and 28 is the presence of a Δ^24(25)^ or Δ^24(28)^ double bond (Nes, 2003), alternative routes may be possible from other precursors having those double bonds (dotted arrows on Figure 2). Finally, to explain the observation of 24-ethylcholest-22-enol in some brown algae, it is necessary to infer the presence of 24-ethylcholesterol as an intermediate, because in that case the lack of Δ^24(25)^ or Δ^24(28)^ would render impossible a sterol-28-methylation from 24-methylcholest-22-enol. Actually, 24-ethylcholesterol has been reported in other brown algae by different groups (Kamenarska et al., 2003; Lopes et al., 2011; Mikami et al., 2018).

Most of the enzymes involved in the sterol biosynthesis pathway are globally conserved across eukaryotes (Supplementary Table S6), but there are some notable exceptions. The sterone-3-reductase performing the third step in the sterol-4-demethylation is known only in animals and fungi (Desmond and Gribaldo, 2009), and its absence in brown algae is consistent with the previously reported absence in plants. The canonical sterol-Δ^24^-reductase and sterol-22-desaturases were lost in brown algae and were most likely replaced by paralogs, given that the metabolic profiling data indicate that those reactions occur (Figure 2). The canonical sterol-4-methyloxidase is found only in *E. subulatus* and in the transcriptome of a fucale, *Sargassum vulgare*. This suggests that this gene may be present in brown algae, but that it is currently unpredicted in the other brown algal genomes due to assembly issues. Altogether, those genomic data indicate that, as for other lineages, the sterol synthesis pathways in brown algae show both conservation and variation regarding other eukaryotic lineages.

### The Late SSR Pathway as a Starting Point for the Biosynthesis of Specialized Metabolites

**Figure 3:**
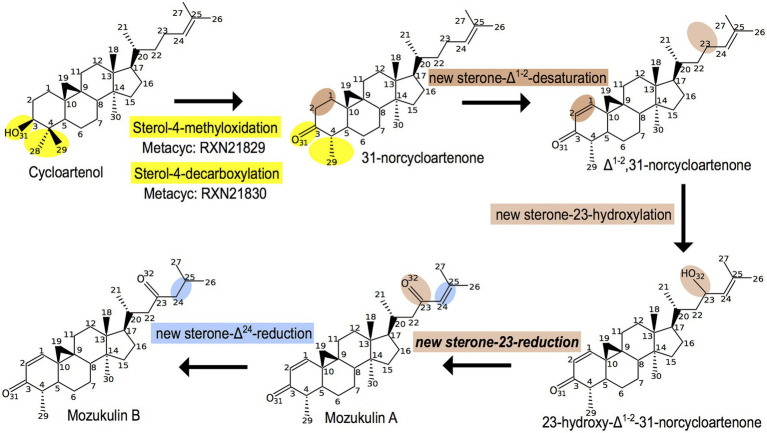
*Ab initio* inference of the mozukulin pathway in the Ectocarpale alga *Cladosiphon okamuranus*. In yellow: reactions identical to the late sterol side-chain reductase (SSR) biosynthesis pathway. In blue: new variant from the Δ^24^-reduction, distinct from those present in the early or late sterol biosynthesis pathways. In brown: reactions variants coming either from other pathways (bacterial steroid degradation or brassinosteroid synthesis) or even more distant type of molecular transformation, previously defined as “type hIIc homologies” (Cunchillos and Lecointre, 2007).

In addition to the sterols that are widespread across brown algae, some derived metabolites of this biosynthetic pathway seem to be restricted to certain species. This is the case of one of the algae for which, we previously performed a GSMN reconstruction (Nègre et al., 2019), the Japanese mozuku *C. okamuranus*. Two sterol derivatives, named mozukulin A and mozukulin B, have been identified in this species (Cheng et al., 2016). In the purpose of globally updating the sterol-related pathway in the GSMN, we also inferred *ab initio* a metabolic pathway model for those molecules using Pathmodel. For this, we could use some molecular transformations already present in the late SSR pathway (in yellow and blue on Figure 3) but also needed to incorporate three other reactions (in brown on Figure 3). The first one, a sterone-Δ^1(2)^-desaturation, has been described in bacteria degrading human sterols in sewage water sediments (Rohman and Dijkstra, 2019), fungi (Lv et al., 2017), and animals (Mahanti et al., 2014). The second one, a sterone-23-hydroxylation, comes from land plant brassinosteroid synthesis (Bajguz et al., 2020). Those two reactions could be easily inferred based on the knowledge from other organisms by the molecular transformation approach as implemented in Pathmodel. For the third reaction, a sterone-23-reduction, we did not find any other similar reactions either in databases or even using classical bibliographic search. However, reduction of hydroxylated carbon residues occurs at various other positions in the sterol backbone: this has been documented on carbons 3, 7, 11, 12, 17, and 20 (Caspi et al., 2020). In that case, the inference is based on a more relaxed concept of molecular transformations (Cunchillos and Lecointre, 2007). We previously justified its use from a biological viewpoint on sterols (Markov et al., 2017), but did not yet implement in Pathmodel. Finally, the sterone-Δ^24^-reduction of Mozukulin A into Mozukulin B is a new variant of reactions observed at the end of the general brown algal sterol biosynthesis pathway (compare with Figure 2). Thus, the first sterol-4-demethylation steps of the late SSR could serve as a branching point for new enzymatic activities leading to the production of specialized molecules. The brown alga-specific sterol-Δ^24^ -reductase, distinct from the ancestral eukaryotic one, may have also been recruited into this pathway due to catalytic promiscuity.

### Integrating Additional Brown Algal Metabolic Profiling Data to Inform Decisions About Genome-Scale Metabolic Network Curation

**Figure 4:**
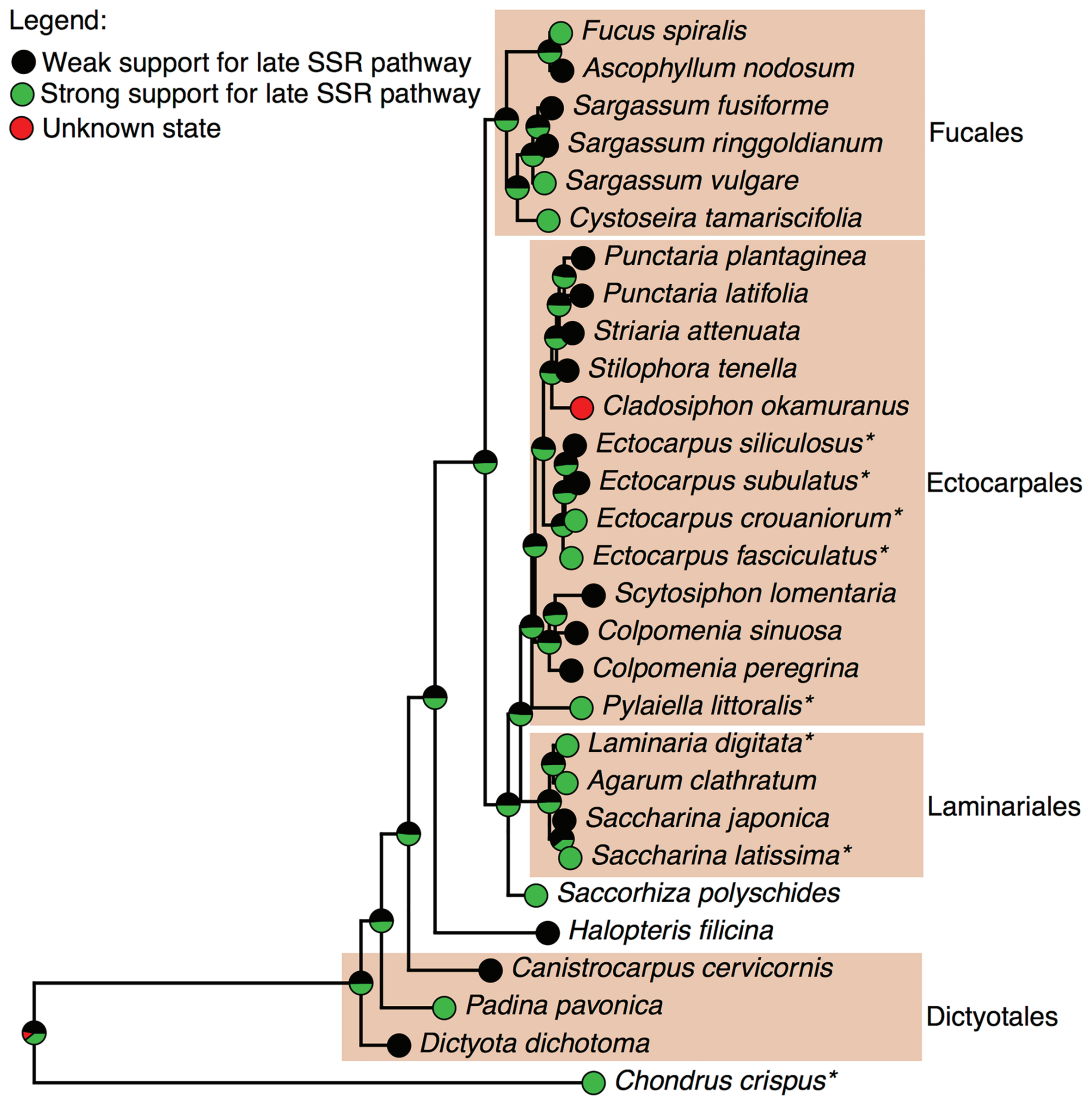
Mapping the degree of support for the late SSR cholesterol pathway in brown algae. The maximum-likelihood tree was inferred based on publicly available data of the *cox3* mitochondrial marker. The four color boxes indicate algal orders where multiple species were analyzed. Strong support: cholesterol and desmosterol detected. Weak support: only cholesterol detected. ^*^represents species with GC-MS data reported in this paper.

The four brown algae for which, we already reconstructed a GSMN illustrate well the classical challenge of heterogeneous data integration. For the two *Ectocarpus* species, we had GC-MS profiling data, partially overlapping, but also partially contradicting those published by others (Mikami et al., 2018). For *S. japonica*, the published sterol profiles (Honya et al., 1994) were in agreement with our own observations in the closely related *S. latissima*. However for *C. okamuranus*, we only had data on specialized metabolites (mozukulins). In order to make a curation decision as well informed as possible, we decided to integrate our profiling results with the rest of the available knowledge on brown algal sterol profiling, which we gathered into an internal database (Supplementary Table S7). There is no experimental support yet for extending the distribution of the mozukulin pathway, for which evidence is up to now limited to *C. okamuranus*. On the contrary, for the fucosterol pathway, there is no reason to doubt about its presence in *C. okamuranus*, because fucosterol is systematically identified in brown algal sterol profiling. However, for the late SSR pathway, the 24-methyl-cholest-22-enol pathway and the pathway from fucosterol to 24-ethyl-cholest-22-enol, the distribution of sterols across species was much variable. This is illustrated with the late SSR pathway in Figure 4, where the data in favor of its wide distribution across brown algae are the strongest among those three pathways.

The topology of this *cox3*-based tree reflects well the current knowledge of phylogenetic relationships among crown brown algae (Bringloe et al., 2020). However, the four Dictyotales species at the basis of the brown algal group (*Dictyota dichotoma*, *Padina pavonica*, *Canistrocarpus cervicornis* and *Halopteris filicina*) do not cluster together, which we interpret as a technical artifact, leading to the overweighting of weak evidence for the late SSR pathway at the ancestral node. Even with this limitation, the probability that the late SSR pathway was present in the common ancestor of Laminariales and Ectocarpales with strong support is 51%. This probability stays identical for the last common ancestor of *C. okamuranus* and the *Punctaria*, *Striata*, and *Stilophora* genera, all corresponding to the Chordariaceae family (Silberfeld et al., 2014). However, because desmosterol has so far not been detected in any species of this group, we decided not to include yet a late SSR pathway in the genome-scale metabolic network for *C. okamuranus*.

## Discussion

In this study, we performed targeted profiling of 12 sterols in 11 algal species and integrated our findings regarding brown algae into biochemical pathway models. We establish a general model for the biosynthesis of the sterols that are widespread across brown algae, but also for some specialized metabolites that are derived from those general pathways. Our analysis therefore provides a starting point for biochemical tests of candidate enzymatic activities.

In addition to the biochemical validation, there are still some issues to further clarify from the metabolomic viewpoint. In particular, there are very few data, to date, on the biosynthetic intermediates between cycloartenol and end-pathway products that we reported here. The only robust evidence here is available for the presence of 24-methylenecholesterol, which was reported in at least 11 other brown algal species (Supplementary Table S7). Those transient compounds are indeed difficult to detect because their abundance is very low in normal physiological states. However, evidence is accumulating in animals, fungi, and land plants that some of them are critical regulators of sterol homeostasis (Darnet and Schaller, 2019). Regarding the end-product sterols, we have to note that although we systematically found 24-methylcholesterol, it was only rarely reported by other studies in different species (Supplementary Table S7), while others reported 24-ethylcholesterol in brown algae, which we found only in the three red algae. This variability is similar to the one found in diatoms: while dihydrobrassicasterol, fucosterol, and clionasterol have been identified in the diatoms *Skeletonema marinoi* and *Cyclotella criptica*, the most abundant sterol in the lipid droplet of the diatom *Phaeodactylum tricornutum* was found to be brassicasterol (Lupette et al., 2019). In addition to the interspecies variations, other factors, like seasonality, are also known to influence the sterol profile variability (Honya et al., 1994). This probably contributed to the variability that we observed across the four *Ectocarpus* species.

Even if important biological variability could impact both quality and quantity of sterols in brown algae, some general patterns, either conserved or not, can be drawn, in relationships with evolution of this pathway. When comparing diatoms and brown algae, which belong both to the stramenopile lineage, it is interesting to note that a pathway identical to the late SSR pathway has been independently proposed (Gallo et al., 2020), the only difference with the late SSR pathway from red algae being that sterol-Δ^8^-Δ^7^ isomerization occurs before the second sterol-4-demethylation, leading to the production of zymosterol. Consistently with this, we inferred the same reaction order for the sterol-Δ^8^-Δ^7^ isomerization and sterol-4-demethylation occurring on methylated sterols, in contrast with the inferences in diatoms that keep the plant-like sterol-4-demethylation before the isomerization step. Regarding this, we have to stress that both hypotheses are equally speculative in light of data about the corresponding biosynthetic intermediates. Another difference between diatoms and brown algae is that some diatoms still retain the canonical SSR enzyme that has been lost in brown algae, despite the evidence that steroid side-chain reduction at carbon 24 does occur in brown algae, according to our profiling data and to independent NMR-based data on specialized metabolites in *C. okamuranus* (Cheng et al., 2016). This observation further supports the hypothesis of metabolic pathway drift by enzymatic replacement, the fact that a pathway can be maintained even if some enzymes are replaced by others over time (Belcour et al., 2020).

## Conclusion

In this paper, we gathered GC-MS data from representatives of two brown algal orders to fuel a first model of sterol biosynthesis pathways and provide a case-study example of how to integrate these data with the literature in an evolutionary context. Specifically, we show that although the most likely way to synthesize cholesterol implies a late SSR pathway, this pathway shows variation either in terms of involved enzymes or in the succession of steps, highlighting the need for experimental data in multiple species to better understand the dynamics of metabolic pathway evolution across the eukaryotic tree of life.

## Data Availability Statement

Data to construct the brown algal biosynthesis pathway: https://github.com/pathmodel/pathmodel/blob/master/pathmodel/data/brown_sterols_pwy.lp. Data to construct the mozukulin biosynthesis pathway in *C. okamuranus*: https://github.com/pathmodel/pathmodel/blob/master/pathmodel/data/mozukulins_pwy.lp. Commands and sources files for ancestral mapping: https://github.com/gabrielmarkov/AncCharMapping. Updated versions of the brown algal genome-scale metabolic networks: http://aureme.genouest.org/wiki.html. All other original contributions presented in the study are included in the article/Supplementary Material; further inquiries can be directed to the corresponding author.

## Author Contributions

GM, LD, SD, CB, CLb, AS, and JC conceived the project. JGi conducted sterol profiling with help from GM, GL, LD, CLr, and CLb. GM encoded the mozukulin, brown algal biosynthesis pathways into the Pathmodel program with input from AB and AS, and wrote the manuscript with input and edits from all authors. LD performed reciprocal blast searches of orthologs in the sterol pathway. JGo updated the genome-scale metabolic network models on the AuReMe website based on input files prepared by GM. All authors contributed to the article and approved the submitted version.

### Conflict of Interest

The authors declare that the research was conducted in the absence of any commercial or financial relationships that could be construed as a potential conflict of interest.

## References

[ref1] Aite M. Chevallier M. Frioux C. Trottier C. Got J. Cortés M. P.. (2018). Traceability, reproducibility and wiki-exploration for “à-la-carte” reconstructions of genome-scale metabolic models. PLOS Comp. Biol. 14, 1–25. 10.1371/journal.pcbi.1006146, PMID: 29791443 PMC5988327

[ref2] Bajguz A. Chmur M. Gruszka D. (2020). Comprehensive overview of the brassinosteroid biosynthesis pathways: substrates, products, inhibitors, and connections. Front. Plant Sci. 11:1034. 10.3389/fpls.2020.01034, PMID: 32733523 PMC7358554

[ref3] Belcour A. Girard J. Aite M. Delage L. Trottier C. Marteau C.. (2020). Inferring biochemical reactions and metabolite structures to understand metabolic pathway drift. iScience 23:100849. 10.1016/j.isci.2020.100849, PMID: 32058961 PMC6997860

[ref4] Bernard M. Rousvoal S. Jacquemin B. Ballenghien M. Peters A. F. Leblanc C. (2018). qPCR-based relative quantification of the brown algal endophyte *Laminarionema elsbetiae* in *Saccharina latissima*: variation and dynamics of host-endophyte interactions. J. Appl. Phycol. 30, 2901–2911. 10.1007/s10811-017-1367-0, PMID: 30416259 PMC6208874

[ref5] Bold H. C. Wynne M. J. (1978). Introduction to the Algae. Englewood Cliffs. New Jersey: Prentice-Hall, xiv+706.

[ref6] Bringloe T. T. Starko S. Wade R. M. Vieira C. Kawai H. de Clerck O.. (2020). Phylogeny and evolution of the brown algae. Crit. Rev. Plant Sci. 39, 281–321. 10.1080/07352689.2020.1787679

[ref7] Caspi R. Billington R. Keseler I. M. Kothari A. Krummenacker M. Midford P. E.. (2020). The MetaCyc database of metabolic pathways and enzymes - a 2019 update. Nucleic Acids Res. 48, D445–D453. 10.1093/nar/gkz862, PMID: 31586394 PMC6943030

[ref8] Cheng K. C. Kuo P. C. Hung H. Y. Yu K. H. Hwang T. L. Shieh P. C.. (2016). Four new compounds from edible algae *Cladosiphon okamuranus* and *Chlorella sorokiniana* and their bioactivities. Phytochem. Lett. 18, 113–116. 10.1016/j.phytol.2016.09.008

[ref9] Cunchillos C. Lecointre G. (2007). Ordering events of biochemical evolution. Biochimie 89, 555–573. 10.1016/j.biochi.2006.12.007, PMID: 17408843

[ref10] Darnet S. Schaller H. (2019). Metabolism and biological activities of 4-methyl-sterols. Molecules 24:451. 10.3390/molecules24030451, PMID: 30691248 PMC6385002

[ref11] Desmond E. Gribaldo S. (2009). Phylogenomics of sterol synthesis: insights into the origin, evolution, and diversity of a key eukaryotic feature. Genome Biol. Evol. 1, 364–381. 10.1093/gbe/evp036, PMID: 20333205 PMC2817430

[ref12] Dittami S. M. Corre E. Brillet-Guéguen L. Lipinska A. P. Pontoizeau N. Aite M.. (2020). The genome of *Ectocarpus subulatus* – A highly stress-tolerant brown alga. Mar. Genomics 52:100740. 10.1016/j.margen.2020.100740, PMID: 31937506

[ref14] Gallo C. Landi S. d’Ippolito G. Nuzzo G. Manzo E. Sardo A.. (2020). Diatoms synthesize sterols by inclusion of animal and fungal genes in the plant pathway. Sci. Rep. 10:4204. 10.1038/s41598-020-60993-5, PMID: 32144288 PMC7060231

[ref39] Gouy M. Guindon S. Gascuel O. (2010). SeaView version 4: a multiplatform graphical user interface for sequence alignment and phylogenetic tree building. Mol. Biol. Evol. 27, 221–224. 10.1093/molbev/msp259 19854763

[ref15] Grebenok R. J. Ohnmeiss T. E. Yamamoto A. Huntley E. D. Galbraith D. W. Della Penna D. (1998). Isolation and characterization of an *Arabidopsis thaliana* C-8,7 sterol isomerase: functional and structural similarities to mammalian C-8,7 sterol isomerase/emopamil-binding protein. Plant Mol. Biol. 38, 807–815. 10.1023/A:1006028623875, PMID: 9862498

[ref40] Guindon S. Gascuel O. (2003). A simple, fast, and accurate algorithm to estimate large phylogenies by maximum likelihood. Syst. Biol. 52, 696–704. 10.1080/10635150390235520 14530136

[ref16] Hamdy A. H. A. Aboutabl E. A. Sameer S. Hussein A. A. Diaz-Marrero A. R. Darias J.. (2009). M. 3-Keto-22-epi-28-nor-cathasterone, a brassinosteroid-related metabolite from *Cystoseira myrica*. Steroids 74, 927–930. 10.1016/j.steroids.2009.06.008, PMID: 19576917

[ref17] Honya M. Kinoshita T. Ishikawa M. Mori H. Nisizawa K. (1994). Seasonal variation in the lipid content of cultured *Laminaria japonica*: fatty acids, sterols, β-carotene and tocopherol. J. Appl. Phycol. 6, 25–29. 10.1007/BF02185900

[ref18] Huelsenbeck J. P. Nielsen R. Bollback J. P. (2003). Stochastic mapping of morphological characters. Syst. Biol. 52, 131–158. 10.1080/10635150390192780, PMID: 12746144

[ref19] Kamenarska Z. G. Dimitrova-Konaklieva S. D. Stefanov K. L. Popov S. S. (2003). A comparative study on the sterol composition of some brown algae from the Black Sea. J. Serb. Chem. Soc. 68, 269–275. 10.2298/JSC0305269K

[ref20] Khoury S. Canlet C. Lacroix M. Z. Berdeaux O. Jouhet J. Bertrand-Michel J. (2018). Quantification of lipids: model, reality, and compromise. Biomol. Ther. 8:174. 10.3390/biom8040174, PMID: 30558107 PMC6316828

[ref21] Lopes G. Sousa C. Bernardo J. Andrade P. B. Valentão P. Ferreres F.. (2011). Sterol profiles in 18 macroalgae of the portuguese coast. J. Phycol. 47, 1210–1218. 10.1111/j.1529-8817.2011.01028.x, PMID: 27020199

[ref22] Lovato M. A. Hart E. A. Segura M. J. Giner J. L. Matsuda S. P. (2000). Functional cloning of an *Arabidopsis thaliana* cDNA encoding cycloeucalenol cycloisomerase. J. Biol. Chem. 275, 13394–13397. 10.1074/jbc.275.18.13394, PMID: 10788449

[ref23] Lupette J. Jaussaud A. Seddiki K. Morabito C. Brugière S. Schaller H.. (2019). The architecture of lipid droplets in the diatom *Phaeodactylum tricornutum*. Algal Res. 38:101415. 10.1016/j.algal.2019.101415

[ref24] Lv J.-M. Hu D. Gao H. Kushiro T. Awakawa T. Chen G.-D.. (2017). Biosynthesis of helvolic acid and identification of an unusual C-4-demethylation process distinct from sterol biosynthesis. Nat. Commun. 8:1644. 10.1038/s41467-017-01813-9, PMID: 29158519 PMC5696383

[ref25] Mahanti P. Bose N. Bethke A. Judkins J. C. Wollam J. Dumas K. J.. (2014). Comparative metabolomics reveals endogenous ligands of DAF-12, a nuclear hormone receptor, regulating *C. elegans* development and lifespan. Cell Metab. 19, 73–83. 10.1016/j.cmet.2013.11.024, PMID: 24411940 PMC3924769

[ref26] Markov G. V. Girard J. Laudet V. Leblanc C. (2018). Hormonally active phytochemicals from macroalgae: a largely untapped source of ligands to deorphanize nuclear receptors in emerging marine animal models. Gen. Comp. Endocrinol. 265, 41–45. 10.1016/j.ygcen.2018.06.004, PMID: 29908834

[ref27] Markov G. V. Gutierrez-Mazariegos J. Pitrat D. Billas I. M. L. Bonneton F. Moras D.. (2017). Origin of an ancient hormone/receptor couple revealed by resurrection of an ancestral estrogen. Sci. Adv. 3:e1601778. 10.1126/sciadv.1601778, PMID: 28435861 PMC5375646

[ref28] Mikami K. Ito M. Taya K. Kishimoto I. Kobayashi T. Itabashi Y.. (2018). Parthenosporophytes of the brown alga *Ectocarpus siliculosus* exhibit sex-dependent differences in thermotolerance as well as fatty acid and sterol composition. Mar. Environ. Res. 137, 188–195. 10.1016/j.marenvres.2018.02.003, PMID: 29459067

[ref29] Nègre D. Aite M. Belcour A. Frioux C. Brillet-Guéguen L. Liu X.. (2019). Genome-scale metabolic networks shed light on the carotenoid biosynthesis pathway in the brown algae *Saccharina japonica* and *Cladosiphon okamuranus*. Antioxidants 8:564. 10.3390/antiox8110564, PMID: 31744163 PMC6912245

[ref30] Nes W. (2003). Enzyme mechanisms for sterol C-methylations. Phytochemistry 64, 75–95. 10.1016/S0031-9422(03)00349-2, PMID: 12946407

[ref42] Nes W. D. (2011). Biosynthesis of Cholesterol and Other Sterols. Chem. Rev. 111, 6423–6451. 10.1021/cr200021m 21902244 PMC3191736

[ref31] Paik Y. K. Trzaskos J. M. Shafiee A. Gaylor J. L. (1984). Microsomal enzymes of cholesterol biosynthesis from lanosterol. Characterization, solubilization, and partial purification of NADPH-dependent delta 8,14-steroid 14-reductase. J. Biol. Chem. 259, 13413–13423. 10.1016/S0021-9258(18)90710-6, PMID: 6444198

[ref32] Rahier A. Bergdoll M. Génot G. Bouvier F. Camara B. (2009). Homology modeling and site-directed mutagenesis reveal catalytic key amino acids of 3beta-hydroxysteroid-dehydrogenase/C4-decarboxylase from *Arabidopsis*. Plant Physiol. 149, 1872–1886. 10.1104/pp.108.132282, PMID: 19218365 PMC2663740

[ref41] R Core Team (2015). R: A language and environment for statistical computing. R Foundation for Statistical Computing, Vienna, Austria. Available at: https://www.R-project.org/

[ref33] Revell L. J. (2012). Phytools: an R package for phylogenetic comparative biology (and other things). Methods Ecol. Evol. 3, 217–223. 10.1111/j.2041-210X.2011.00169.x

[ref34] Rohman A. Dijkstra B. W. (2019). The role and mechanism of microbial 3-ketosteroid Δ(1)-dehydrogenases in steroid breakdown. J. Steroid Bioch. 191:105366. 10.1016/j.jsbmb.2019.04.015, PMID: 30991094

[ref35] Rubinstein I. Goad L. Clague A. Mulheirn L. J. (1976). The 220 MHz NMR spectra of phytosterols. Phytochemistry 15, 195–200. 10.1016/S0031-9422(00)89083-4

[ref36] Sievers F. Higgins D. G. (2014). Clustal omega, accurate alignment of very large numbers of sequences. Methods Mol. Biol. 1079, 105–116. 10.1007/978-1-62703-646-7_6, PMID: 24170397

[ref37] Silberfeld T. Rousseau F. de Reviers B. (2014). An updated classification of brown algae (Ochrophyta, Phaeophyceae). Cryptogam. Algol. 35, 117–156. 10.7872/crya.v35.iss2.2014.117

[ref38] Sonawane P. D. Pollier J. Panda S. Szymanski J. Massalha H. Yona M.. (2016). Plant cholesterol biosynthetic pathway overlaps with phytosterol metabolism. Nat. plants 3:16205. 10.1038/nplants.2016.205 28005066

