# Supplementary material for: Semi-Quantitative Targeted Gas Chromatography-Mass Spectrometry Profiling Supports a Late Side-Chain Reductase Cycloartenol-to-Cholesterol Biosynthesis Pathway in Brown Algae
Source: Front Plant Sci. 2021 Apr 27;12:648426. doi: 10.3389/fpls.2021.648426 (PMC8112355; doi:10.3389/fpls.2021.648426)
Supplement: Supplementary file 1 [file Data_Sheet_1.PDF]

Supplementary Material

Semi-quantitative targeted GC-MS profiling supports a late side-chain reductase cycloartenol-to-cholesterol biosynthesis pathway in brown algae

Jean Girard[1], Goulven Lanneau[1,2†], Ludovic Delage[1], Cédric Leroux[2], Arnaud Belcour[3], Jeanne Got[3], Jonas Collén[1], Catherine Boyen[1], Anne Siegel[3], Simon M. Dittami[1], Catherine Leblanc[1], Gabriel V. Markov[1*]

[1]Sorbonne Université, CNRS, Integrative Biology of Marine Models (LBI2M, UMR8227), Station Biologique de Roscoff (SBR), 29680 Roscoff, France

[2] Sorbonne Université, CNRS, Plateforme Corsaire-METABOMER (FR2424), Station Biologique de Roscoff, Roscoff, France

[3]Univ Rennes, Inria, CNRS, IRISA, Equipe Dyliss, Rennes, France

[†] Deceased

*Corresponding author, Lead Contact

Email: Gabriel.Markov@sb-roscoff.fr

Sterol quantification protocol

We first tried to work on Selected Ion Monitoring (SIM) mode, but the starting time and the quantitative ions were the same, or very closed for some of our sterols, following to not retain this classical approach. So we have focused on Internal Standard corrections. We compared the standard curves corrected by three different ways. The first is a correction on individual standard, used for simple lipid where calibration curves can be performed to correct detector sensitivity. The second is a correction on a global mix standards, closer to real complex solution of our samples. The last is a correction on average standard curves between mix standards which contained all sterols of the global mix standards, but divided in two new mixes with time retention well distinct. All standards were used as tetramethylsilane (TMS) derivatives.

SIM method

The SIM method is classically useful to quantify compound in GC-MS. Here we give the different parameters that we used and the chromatograms of mix standards and algae extract obtained with this SIM method.

Start time	Default	Group	Target ion	Target ion	Target ion	Target ion	Target ion	Sterols
0	100	1	74.10					
19.50	25	2	69	81	95	136	137	Squalene
20	25	3	217	357	372	149	109	Cholestane
24.50	25	4	329	129	368	458	353	Cholesterol (1)
25.15	25	5	351	343	325	129	69	Brassicasterol (2)
25.30	25	5	129	69	255	380	470	Desmosterol (3)
25.35	25	5	351	325	366	143	73	7 dehydrocholesterol (4)
25.61	25	5	458	456	255	107	75	Lathosterol (5)
25.70	25	5	456	441	351	213	69	Zymosterol (6)
26	25	7	363	337	81	73	69	Ergosterol (7)
26.30	25	8	343	129	382	472	367	Campesterol (8)
26.80	25	9	83	129	255	394	484	Stigmasterol (9)
27.50	25	10	393	69	498	483	109	Lanosterol (10)
27.90	25	11	357	129	396	486	381	β-sitosterol (11)
27.95	25	11	386	357	129	73	296	Fucosterol (12)
28.70	25	12	408	393	365	69	95	Cycloartenol (13)
30.10	25	13	189	73	109	95	190	Cycloeucalenol (14)
30.48	25	14	95	109	123	185	237	Cycloartanol (15)
31.10	25	15	95	69	107	81	422	24 methylene cycloartanol (16)

Table S1 : retention time, repartition by group and highest target ions (dwell = 0.5) used for each standard in the mix.

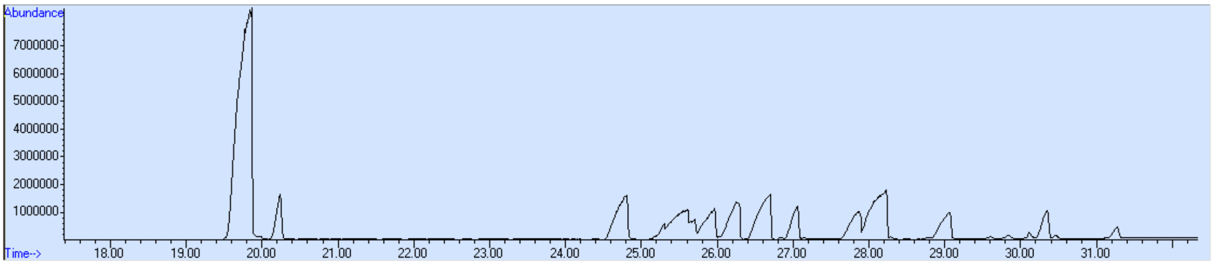

Figure S1 : Chromatogram obtained with the Table 1 SIM parameters (200ng of each standard was injected).

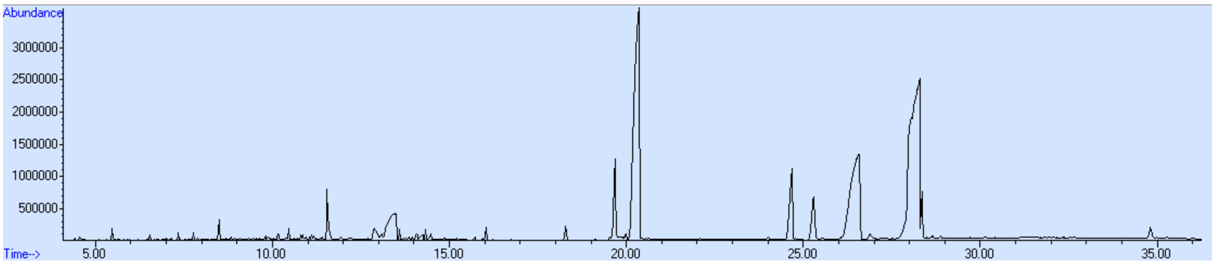

Figure S2: Chromatogram obtained with the Table 1 SIM parameters (algal extract: *Ectocarpus fasciculatus*).

On Figures S1 and S2, peaks are always at low intensity and co-eluted sterols could not be detected. Moreover, on Figure S2, all the fucosterol was not detected because it appears before the start time. The SIM method necessitates a very good separation between consecutive peaks, that is not the case in this study. The «start time» parameter does not allow to detect the totality of a compound present in high quantity like fucosterol in our algal extracts. Therefore, the SIM method was not appropriate for our study, we focused on Internal Standard method for relative quantification.

Cholestane corrections

The previous method based on the area of the internal standard, that we will call without correction.

Internal standard = cholestane (5 µg by sample corresponding to 50 ng injected by GC-MS)

The quantity of each sterol (in µg) is: Area of the standard * 5 / Area of cholestane.

This method is useful for a study on a single sterol, but it introduce some bias in complex solution like our algae extract. But we keep this method to compare with other methods.

Global mix standards solution with internal standard correction

In complex mixture, the quantitative determination is different from a single standard correction. So, we pooled all standards that we had to create a global standards mix, called Mix 1.

To quantify sterols, we prepared a calibration range (Table S2).

**Volume of mix standard (m=100 µg) withdrawn (µl)**	**400**	**350**	**300**	**250**	**200**	**150**	**100**	**50**	**20**	**0**
**masse of sterol in the mix to analyse (µg)**	20	17.5	15	12.5	10	7.5	5	2.5	1	0
**Injected mass (ng)**	200	175	150	125	100	75	50	25	10	0
**+**	5 µl of cholestane (50 ng injected)									

Table S2 : Preparation of standards range from 0 to 20 µg : Mix standard 1 (global standards mix).

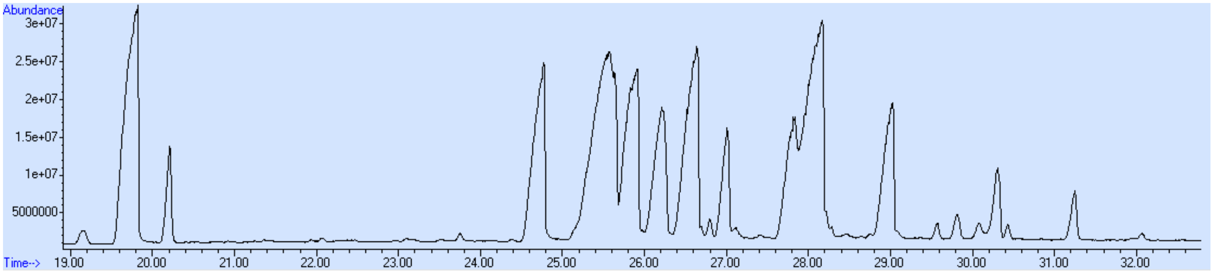

Figure S3 : chromatogram of Mix standard 1 (global standards mix). Co-elution of some standards, impossible to quantify.

Dividing the global mix to separate the sterols who co-eluted

We created two new mixes without col-eluted sterols for a better determination of individual peaks, being in a « semi-complex » system (Figures S4 and S5).

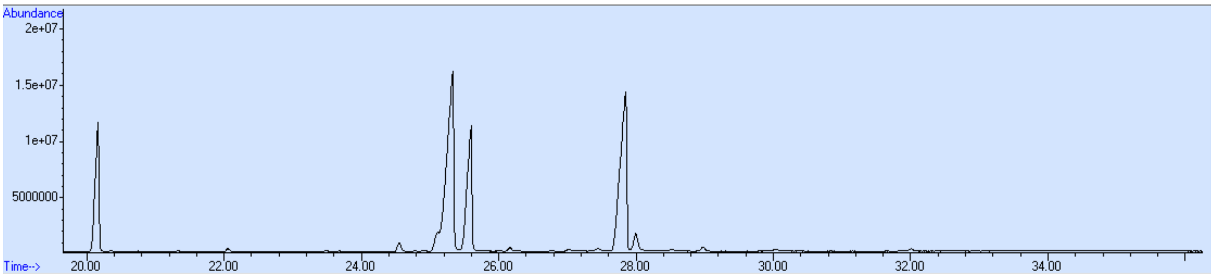

Figure S4 : Mix standard 2 : range of dilution from 0 to 20 µg with desmosterol, lathosterol and fucosterol.

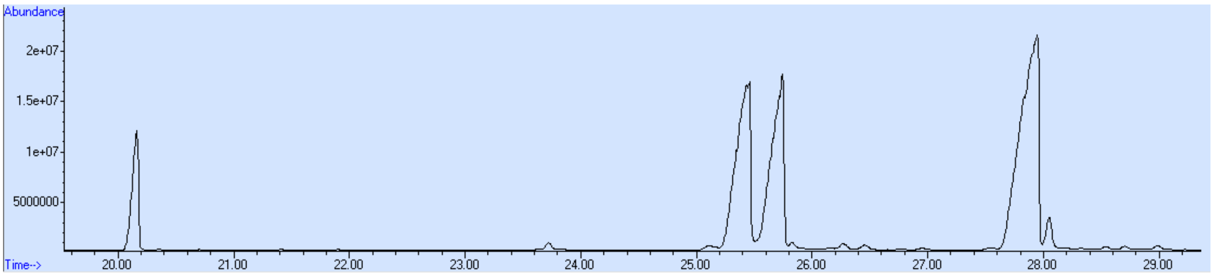

Figure S5 : Mix standard 3 : range of dilution from 0 to 20 µg with brassicasterol, zymosterol and β-sitosterol.

Equations and Corrections tested

Correction 1 :

Corrected area of a peak = (Area $_{\text{average cholestane on 3 mix}}$ /Area $_{\text{cholestane}}$) * Aera of peak

Correction 2 :

Corrected area of a peak = (Aire $_{\text{avrage cholestane on mix}}$ /Area $_{\text{cholestane}}$) * Area of peak

Standard	Mix	No corrected Area		Corrected area n° 1		Corrected area n° 2	
		Equation	R^2	Equation	R^2	Equation	R^2
Squalene	1	y = 1.89E+07x	0.960	y = 1.63E+07x	0.997	y = 2.17E+07x	0.997
Cholesterol	1	y = 1.19E+07x	0.979	y = 1.03E+07x	0.999	y = 1.37E+07x	0.999
Desmosterol	2	y = 5.59E+06x	1	y = 6.54E+06x	0.996	y = 5.56E+06x	0.996
Brassicasterol	3	y = 7.06E+06x	0.998	y = 8.25E+06x	0.984	y = 6.76E+06x	0.984
Lathosterol	2	y = 2.69E+06x	1	y = 3.15E+06x	0.995	y = 2.68E+06x	0.995
Zymosterol	3	y = 6.38E+06x	0.998	y = 7.45E+06x	0.984	y = 6.10E+06x	0.984
Ergosterol	1	y = 9.50E+06x	0.976	y = 8.22E+06x	0.999	y = 1.09E+07x	0.999
Campesterol	1	y = 1.50E+07x	0.966	y = 1.29E+07x	0.998	y = 1.72E+07x	0.998
Stigmasterol	1	y = 4.83E+06x	0.976	y = 4.18E+06x	0.999	y = 5.55E+06x	0.999
Lanosterol		y = 8.98E+06x	0.955	y = 7.75E+06x	0.995	y = 1.03E+07x	0.995
Fucosterol	2	y = 4.62E+06x	0.999	y = 5.40E+06x	0.995	y = 4.59E+06x	0.995
β-sitosterol	3	y = 1.15E+07x	0.997	y = 1.34E+07x	0.984	y = 1.10E+07x	0.984
Cycloartenol	1	y = 8.39E+06x	0.976	y = 7.26E+06x	0.999	y = 9.65E+06x	0.999
Cycloeucalenol	1	y = 3.10E+06x	0.977	y = 2.69E+06x	0.996	y = 3.57E+06x	0.996
Cycloartanol	1	y = 515296x	0.860	y = 442840x	0.929	y = 588994x	0.9289
24 methylene cycloartenol	1	y = 1.78E+06x	0.969	y = 1.54E+06x	0.989	y = 2.05E+06x	0.989

Table S3 : Equation of calibration curves depending of the correction applied, for each sterol.

Sterol	Mass in 60 µg of algae (µg)			
	Method based on cholestane	Range without correction	Range with correction n°1	Range with correction n°2
Squalene	1.403	2.734	3.170	2.381
Cholesterol	1.400	4.334	5.007	3.764
Desmosterol	0.342	2.349	2.009	2.347
Campesterol	0.365	0.897	1.043	0.782
Stigmasterol	0.198	0.487	1.748	1.317
Fucosterol	33.758	264.774	226.281	265.888
Cycloartenol	0.348	1.529	1.767	1.329
Total	37.949	277.653	241.661	278.287

Table S4: Comparaison of quantification methods on the same algal extract (*Ulva sp.*).

Species	Method based on cholestane area	Method based on calibration curve n°1	Method based on calibration curve n°2	Literature
Chondrus crispus	0.18 ± 0.02	0.53 ± 0.05	0.68 ± 0.06	0.18<br>Tasende *et al*, 2000
Palmaria palmata	0.39 ± 0.07	2.34 ± 0.35	1.76 ± 0.25	0.40<br>Gibbons et al, 1967
Ectocarpus siliculosus	0.725 ± 0.04	4.889 ± 0.25	4.437 ± 0.03	4.05 ± 0.95<br>Mikami *et al*, 2018
Ulva sp.	0.58 ± 0.05	4.52 ± 0.54	4.10 ± 0.49	1.40<br>Kendel *et al*, 2015

Table S5 : Quantity of sterols in extracts depending on the method used.

Depending of method used, the total amount of sterol changes in an important way. Except for *Ectocarpus siliculosus*, were the quantification was performed using HPLC coupled to fluorimetry, the method based on cholestane area allows to obtain results found in litterature (Table S5). But, this one is relatively old and the method of quantification used are not mentioned. In our study, we noticed that this method undervalues the quantity of sterols present in the sample in an important way and particulary for the fucosterol. The method with calibration curve n°1 gives a good coefficient of correlation but only for the Mix1. However, the method with calibration curve n°2 gives a good result for all Mixes. This is why we choosed this method, that seems to be more appropriate and accurate than the others, keeping a relative quantitative method (or close quantification with caution).

References

Gibbons, G., Goad, L., and Goodwin, T. (1967). The sterols of some marine red algae. *Phytochemistry 6*, 677-683. doi: 10.1016/S0031-9422(00)86007-0

Kendel, M., Wielgosz-Collin, G., Bertrand, S., Roussakis, C., Bourgougnon, N. et al. (2015). Lipid Composition, Fatty Acids and Sterols in the Seaweeds *Ulva armoricana*, and *Solieria chordalis* from Brittany (France): An Analysis from Nutritional, Chemotaxonomic, and Antiproliferative Activity Perspectives. *Mar. Drugs,* **2015***, 13*, 5606-28. doi: 10.3390/md13095606

Mikami, K., Ito, M., Taya, K., Kishimoto, I., Kobayashi, T., Itabashi, Y. et al. (2018). Parthenosporophytes of the brown alga *Ectocarpus siliculosus* exhibit sex-dependent differences in thermotolerance as well as fatty acid and sterol composition. Mar. Environ. Res. 137, 188-195. doi: 10.1016/j.marenvres.2018.02.003

Tasende, M. (2000). Fatty acid and sterol composition of gametophytes and sporophytes of *Chondrus crispus* (Gigartinaceae, Rhodophyta). Sci. Mar. 64, 421-426. doi: 10.3989/scimar.2000.64n4421

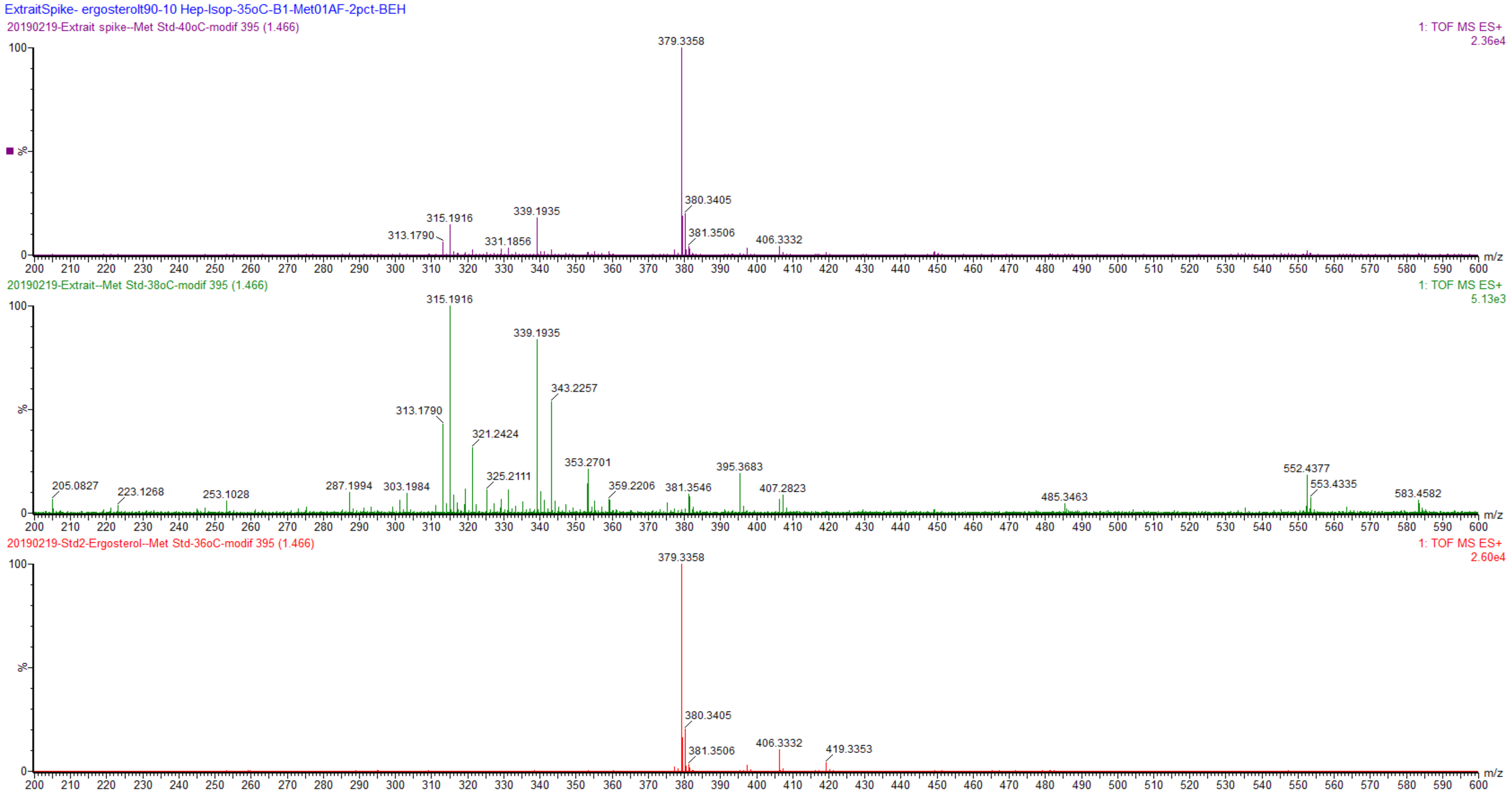

Figure S6. Control for technical detectability of ergosterol in spiked *Ectocarpus siliculosus* extract.
a) MS spectrum from *E. siliculosus* extract incubated with ergosterol. b) MS spectrum of ergosterol standard incorporated in *E. siliculosus* extract. c) MS spectrum of ergosterol standard alone.

Table S6 : main identified orthologs in the sterol pathway for macroalgae

Species	Order	Squalene monooxygenase	Oxidosqualene cyclase	Sterol-14-demethylase	Sterol-14-reductase	Sterol-4-methyloxidase	Sterol-3-dehydrogenase Sterol-4-decarboxylase	Sterone-3-ketoreductase	Sterol-Δ7-Δ8-isomerase	Sterol-5-desaturase	Sterol-24/28-methyltransferase	Sterol-Δ7-reductase	Sterol-Δ24-reductase	Sterol-22-desaturase	Cyclopropylsterol cycloisomerase	Total	Database	Accession
Canistrocarpus cervicornis	*Dictyotales*	nd	nd	nd	nd	nd	nd	nd	nd	nd	nd	nd	nd	nd	nd	nd		
Dictyota dichotoma	*Dictyotales*															10/14	SRA	SRR5088957
Padina pavonica	*Dictyotales*	nd	nd	nd	nd	nd	nd	nd	nd	nd	nd	nd	nd	nd	nd	nd		
Cladosiphon okamuranus	*Ectocarpales*															9/14		
Colpomenia peregrina	*Ectocarpales*	nd	nd	nd	nd	nd	nd	nd	nd	nd	nd	nd	nd	nd	nd	nd		
Colpomenia sinuosa	*Ectocarpales*															10/14	TSA	GFKL
*Ectocarpus crouanorium**	*Ectocarpales*	nd	nd	nd	nd	nd	nd	nd	nd	nd	nd	nd	nd	nd	nd	nd		
*Ectocarpus fasciculatus**	*Ectocarpales*	nd	nd	nd	nd	nd	nd	nd	nd	nd	nd	nd	nd	nd	nd	nd		
*Ectocarpus siliculosus**	*Ectocarpales*															10/14		
*Ectocarpus subulatus**	*Ectocarpales*															10/14		
*Pylaiella littoralis**	*Ectocarpales*	nd	nd	nd	nd	nd	nd	nd	nd	nd	nd	nd	nd	nd	nd	nd		
Punctaria latifolia	*Ectocarpales*															10/14	SRA	SRX2653511
Punctaria plantaginea	*Ectocarpales*	nd	nd	nd	nd	nd	nd	nd	nd	nd	nd	nd	nd	nd	nd	nd		
Scytosiphon lomentaria	*Ectocarpales*															10/14	TSA	GFKH
Stilophora tennella	*Ectocarpales*	nd	nd	nd	nd	nd	nd	nd	nd	nd	nd	nd	nd	nd	nd	nd		
Striaria attenuata	*Ectocarpales*	nd	nd	nd	nd	nd	nd	nd	nd	nd	nd	nd	nd	nd	nd	nd		
Ascophyllum nodosum	*Fucales*															10/14	SRA	ERX2240951
Cystoseira tamariscifolia	*Fucales*	nd	nd	nd	nd	nd	nd	nd	nd	nd	nd	nd	nd	nd	nd	nd		
Fucus spiralis	*Fucales*															10/14	SRA	SRX7730724
Sargassum fusiforme	*Fucales*															10/14	SRA	SRX2653515
Sargassum ringgoldianum	*Fucales*	nd	nd	nd	nd	nd	nd	nd	nd	nd	nd	nd	nd	nd	nd	nd		
Sargassum vulgare	*Fucales*															11/14	TSA	GEHA
Agarum clathratum	*Laminariales*															10/14	TSA	PRJNA320141
*Laminaria digitata**	*Laminariales*															10/14	TSA	PRJNA320141
Saccharina japonica	*Laminariales*															10/14		

*Saccharina latissima**	*Laminariales*															10/14	TSA	PRJNA320141
Halopteris filicina	*Sphacelariales*	nd	nd	nd	nd	nd	nd	nd	nd	nd	nd	nd	nd	nd	nd	nd		
Saccorhiza polyschides	*Tilopteridales*	nd	nd	nd	nd	nd	nd	nd	nd	nd	nd	nd	nd	nd	nd	nd		
Ulva mutabilis	*Ulvales*															10/14		
Ulva sp.	*Ulvales*	nd	nd	nd	nd	nd	nd	nd	nd	nd	nd	nd	nd	nd	nd	nd		
*Chondrus crispus**	*Gigartinales*															11/14		
*Mastocarpus stellatus**	*Gigartinales*	nd	nd	nd	nd	nd	nd	nd	nd	nd	nd	nd	nd	nd	nd	nd		
*Palmaria palmata**	*Palmariales*															12/14	TSA	GFTH

Legend

presence of identified ortholog(s)

absence of identified ortholog(s)

Background colors of the enzyme names correspond to the reactions indicated in Fig. 2.

Species/Orders are in brown, green and red bold polices respectively for brown, green and red macroalgae.

Species with a sterol chemical profiling in this study are indicated by *

nd : not determined

Table S7 : Presence-absence data of sterols in brown algae compiled from previous studies, with accession numbers for available *cox3* sequences.

Species	Order	Desmosterol	Cholesterol	24-methylene-cholesterol	24-methyl-cholesterol	24-methyl-cholest-22-enol	Fucosterol	24-ethyl-cholesterol	24-ethyl-cholest-22-enol	cox3	References
Canistrocarpus cervicornis	Dictyotales	-	x	x	x	x	x	-	x	MF538759.1	Fleury1994
Dictyota dichotoma	Dictyotales	-	x	-	-	-	x	-	x	MK516769.1	Fleury1994
Padina pavonica	Dictyotales	x	x	-	x	-	x	-	x	LN612766.1	Kamenarska2002
Cladosiphon okamuranus	Ectocarpales	?	?	?	?	?	?	?	?	LC016524.1	this study
Colpomenia peregrina	Ectocarpales	-	x	x	-	x	x	-	-	JX027375.1	Kamenarska2003
Colpomenia sinuosa	Ectocarpales	-	x	-	-	-	x	-	x	JX944760.1	Kanias1992
*Ectocarpus crouanorium**	Ectocarpales	x	x	?	x	x	x	-	-	FN564514.1	this study
*Ectocarpus fasciculatus**	Ectocarpales	x	x	?	x	x	x	-	x	FN564518.1	this study
*Ectocarpus siliculosus**	Ectocarpales	-	x	?	x	-	x	x	x	FN564512.1	Mikami2018
*Ectocarpus subulatus**	Ectocarpales	-	-	?	x	-	x	-	-	JN406856.1	this study
*Pylaiella littoralis**	Ectocarpales	x	x	?	x	-	x	-	x	NC_003055.1	this study
Punctaria latifolia	Ectocarpales	-	x	x	-	x	x	x	-	EU681459.1	Kamenarska2003
Punctaria plantaginea	Ectocarpales	-	x	x	-	x	x	-	-	AB302317.1	Kamenarska2003
Scytosiphon lomentaria	Ectocarpales	-	x	x	-	x	x	-	x	NC_025240.1	Kamenarska2003
Stilophora tennella	Ectocarpales	-	x	x	-	-	x	x	-	LC201584.1	Kamenarska2003
Striaria attenuata	Ectocarpales	-	x	x	-	x	x	x	x	LC016538.1	Kamenarska2003
Ascophyllum nodosum	Fucales	-	x	x	-	-	x	-	-	EU681433.1	Knights1970
Cystoseira tamariscifolia	Fucales	x	x	-	-	-	x	x	-	EU681443.1	Lopes2011
Fucus spiralis	Fucales	x	x	-	-	-	x	-	-	MG922856.1	Lopes2011
Sargassum fusiforme	Fucales	-	-	x	-	-	x	-	-	KX085174.1	Chen2014
Sargassum ringgoldianum	Fucales	-	x	x	-	-	x	-	-	KY935448.1	Ikekawa1968
Sargassum vulgare	Fucales	x	x	-	-	-	x	x	-	KJ572507.1	Lopes2011
Agarum clathratum	Laminariales	x	x	x	-	-	x	-	-	HQ871492.1	Newburger1979
*Laminaria digitata**	Laminariales	x	x	x	-	-	x	-	-	AJ344328.1	Patterson1968
Saccharina japonica	Laminariales	-	x	x	-	-	x	-	-	AB775245.1	Honya1994
*Saccharina latissima**	Laminariales	x	x	x	-	-	x	-	-	NC_026108.1	Patterson1968
Halopteris filicina	Sphacelariales	-	x	-	-	-	x	-	-	KJ175242.1	Lopes2011
Saccorhiza polyschides	Tilopteridales	x	x	-	-	-	x	-	-	AB543506.1	Lopes2011
Chondrus crispus	red alga	x	x	?	x	-	-	x	x	NC_001677.2	this study

**Legend**		
	Present :	x
	Not detected:	-
	Unknown :	?

Species with a sterol chemical profiling in this study are indicated by *

NB : for brown algal species profiled also by other authors, the external reference is indicated instead of « this study »

References for Table S7

Chen, Z., Liu, J., Fu, Z., Ye, C., Zhang, R., Song, Y., et al. (2014). 24(S)-Saringosterol from edible marine seaweed *Sargassum fusiforme* is a novel selective LXRβ agonist. J. Agr. Food Chem. 62, 6130-6137. doi: 10.1021/jf500083r

Fleury, B.G., Pereira, M.V., Da Silva, J.R., Kaisin, M., Teixeira, V.L., Kelecom, A. (1994). Sterols from brazilian marine brown algae. Phytochemistry 37, 1447-1449. doi: 10.1016/S0031-9422(00)90430-8

Honya, M., Kinoshita, T., Ishikawa, M., Mori, H., and Nisizawa, K. (1994). Seasonal variation in the lipid content of cultured *Laminaria japonica*: fatty acids, sterols, β-carotene and tocopherol. J. Appl. Phycol. 6, 25-29. doi: 10.1007/BF02185900

Ikekawa, N., Morisaki, N., Tsuda, K., Yoshida, T. (1968). Sterol composition in some green algae and brown algae. Steroids 12, 41-48. doi: 10.1016/S0039-128X(68)80078-9

Kamenarska, Z., Gasic, M.J., Zlatovic, M., Rasovic, A., Sladic, D., Kljajic, Z., et al. (2002). Chemical Composition of the Brown Alga *Padina pavonia* (L.) Gaill. from the Adriatic Sea. Bot. Mar. 45, 339–345. doi: 10.1515/BOT.2002.034

Kamenarska, Z.G., Dimitrova-Konaklieva, S.D., Stefanov, K.L., Popov, S.S. (2003). A comparative study on the sterol composition of some brown algae from the Black Sea. J. Serb. Chem. Soc. 68, 269-275.

Kanias, G.D., Skaltsa, H., Tsitsa, E., Loukis, A., Bitis, J. (1992). Study of the correlation between trace elements, sterols and fatty acids in brown algae from the Saronikos gulf of Greece. Fresenius' J. Anal. Chem. 344, 334-339.

Knights, B. (1970). Sterols in *Ascophyllum nodosum*. Phytochemistry 9, 903 - 905. doi: 10.1016/S0031-9422(00)85204-8

Lopes, G., Sousa, C., Bernardo, J., Andrade, P.B., Valentão, P., Ferreres, F. et al. (2011). Sterol profiles in 18 macroalgae of the portuguese coast. J. Phycol. 47, 1210-1218. doi:10.1111/j.1529-8817.2011.01028.x

Mikami, K., Ito, M., Taya, K., Kishimoto, I., Kobayashi, T., Itabashi, Y. et al. (2018). Parthenosporophytes of the brown alga *Ectocarpus siliculosus* exhibit sex-dependent differences in thermotolerance as well as fatty acid and sterol composition. Mar. Environ. Res. 137, 188-195. doi: 10.1016/j.marenvres.2018.02.003

Newburger, J.D., Uebel, J., Ikawa, M., Andersen, K.K., Gagosian, R.B. (1979). Sterols of *Agarum cribosum*: desmosterol in a brown alga. Phytochemistry 18, 2042-2043. doi: 10.1016/S0031-9422(00)82738-7

Patterson, G.W. (1968). Sterols of *Laminaria*. Comp. Biochem. Physiol. 24, 501-505. doi: 10.1016/0010-406X(68)91001-3

Table S8. List of reactions added in the genome-scale metabolic network of *C. okamuranus*

reaction_id	RXN-21829
comment	Curation Sterols (Girard et al.)
reversible	false
linked_gene	
#reactant/product	#stoichio:compound_id:compart
reactant	1.0:CYCLOARTENOL:c
product	1.0:CPD-23708:c
pathway	PWY-8191

reaction_id	RXN-21830
comment	Curation Sterols (Girard et al.)
reversible	false
linked_gene	
#reactant/product	#stoichio:compound_id:compart
reactant	1.0:CPD-23708:c
product	1.0:CPD-23709:c
pathway	PWY-8191

reaction_id	new_delta1-2_desaturation
comment	pathmodel inference
reversible	false
linked_gene	
#reactant/product	#stoichio:compound_id:compart
reactant	1.0:CPD-23709:c
product	1.0:DELTA1-2,31-NORCYCLOARTENONE:c
pathway	Mozukulin biosynthesis

reaction_id	new_23_hydroxylation
comment	pathmodel inference
reversible	false
linked_gene	
#reactant/product	#stoichio:compound_id:compart
reactant	1.0:DELTA1-2,31-NORCYCLOARTENONE:c
product	1.0:23-HYDROXY-DELTA1-2,31-NORCYCLOARTENONE:c
pathway	Mozukulin biosynthesis

reaction_id	new_23_reduction
comment	pathmodel inference
reversible	false
linked_gene	
#reactant/product	#stoichio:compound_id:compart
reactant	1.0:23-HYDROXY-DELTA1-2,31-NORCYCLOARTENONE:c
product	1.0:MOZUKULIN_A:c
pathway	Mozukulin biosynthesis

reaction_id	new_delta24_reduction
comment	pathmodel inference
reversible	false
linked_gene	
#reactant/product	#stoichio:compound_id:compart
reactant	1.0:MOZUKULIN_A:c
product	1.0:MOZUKULIN_B:c
pathway	Mozukulin biosynthesis

reaction_id	RXN-22206
comment	Gallo2020
reversible	false
linked_gene	
reactant	1.0:CPD-24184:c
product	1.0:CPD-24185:c
pathway	PWY-8238

reaction_id	RXN-22198
comment	Gallo2020
reversible	false
linked_gene	g12612.t1
reactant	1.0:CPD-24185:c
product	1.0:CPD-22095:c
pathway	PWY-8238

Table S9. List of reactions added in the genome-scale metabolic network of *E. siliculosus*

reaction_id	RXN-21829
comment	Curation Sterols (Girard et al.)
reversible	false
linked_gene	
#reactant/product	#stoichio:compound_id:compart
reactant	1.0:CYCLOARTENOL:c
product	1.0:CPD-23708:c
pathway	PWY-8191

reaction_id	RXN-21830
comment	Curation Sterols (Girard et al.)
reversible	false
linked_gene	Esi0147_0062
#reactant/product	#stoichio:compound_id:compart
reactant	1.0:CPD-23708:c
product	1.0:CPD-23709:c
pathway	PWY-8191

reaction_id	RXN-21831
comment	Curation Sterols (Girard et al.)
reversible	false
linked_gene	
#reactant/product	#stoichio:compound_id:compart
reactant	1.0:CPD-23709:c
product	1.0:CPD-12850:c
pathway	PWY-8191

reaction_id	RXN11876
comment	Curation Sterols (Girard et al.)
reversible	false
linked_gene	Esi0169_0047
#reactant/product	#stoichio:compound_id:compart
reactant	1.0:CPD-12850:c
product	1.0:CPD-12852:c
pathway	PWY-8191

reaction_id	RXN11881
comment	Curation Sterols (Girard et al.)
reversible	false
linked_gene	Esi0006_0200
#reactant/product	#stoichio:compound_id:compart
reactant	1.0:CPD-12852:c
product	1.0:CPD-12853:c
pathway	PWY-8191

reaction_id	RXN11878
comment	Curation Sterols (Girard et al.)
reversible	false
linked_gene	Esi0073_0082
#reactant/product	#stoichio:compound_id:compart
reactant	1.0:CPD-12853:c
product	1.0:4-METHYL-824-CHOLESTADIENOL:c
pathway	PWY-8191

reaction_id RXN-13709
comment Curation Sterols (Girard et al.)
reversible false
linked_gene
#reactant/product #stoichio:compound_id:compart
reactant 1.0:4-METHYL-824-CHOLESTADIENOL:c
product 1.0:CPD-4702:c
pathway PWY-8239

reaction_id RXN66-318
comment Curation Sterols (Girard et al.)
reversible false
linked_gene Esi0147_0062
#reactant/product #stoichio:compound_id:compart
reactant 1.0:CPD-4702:c
product 1.0:CPD-4581:c
pathway PWY-8239

reaction_id RXN66-319
comment Curation Sterols (Girard et al.)
reversible false
linked_gene
#reactant/product #stoichio:compound_id:compart
reactant 1.0:CPD-4581:c
product 1.0:ZYMOSTEROL:c
pathway PWY-8239

reaction_id RXN66-320
comment Curation Sterols (Girard et al.)
reversible true
linked_gene Esi0155_0043
#reactant/product #stoichio:compound_id:compart
reactant 1.0:ZYMOSTEROL:c
product 1.0:5-ALPHA-CHOLESTA-724-DIEN-3-BETA-OL:c
pathway PWY-8239

reaction_id RXN-11887
comment Curation Sterols (Girard et al.)
reversible false
linked_gene Esi0003_0314
reactant 1.0:5-ALPHA-CHOLESTA-724-DIEN-3-BETA-OL:c
product 1.0:CPD-8646:c
pathway PWY-8191

reaction_id RXN-22206
comment Gallo2020
reversible false
linked_gene Esi0003_0314
reactant 1.0:CPD-24184:c
product 1.0:CPD-24185:c
pathway PWY-8238

reaction_id RXN-22198
comment Gallo2020
reversible false
linked_gene Esi0044_0110
reactant 1.0:CPD-24185:c
product 1.0:CPD-22095:c
pathway PWY-8238

Table S10. List of reactions added in the genome-scale metabolic network of *E. subulatus*

reaction_id	RXN-21829
comment	Curation Sterols (Girard et al.)
reversible	false
linked_gene	EsuFWS8841_2 and EsuFWS13301_2
#reactant/product	#stoichio:compound_id:compart
reactant	1.0:CYCLOARTENOL:c
product	1.0:CPD-23708:c
pathway	PWY-8191

reaction_id	RXN-21830
comment	Curation Sterols (Girard et al.)
reversible	false
linked_gene	CACKRE030002836.1 and CAB1107767.1
#reactant/product	#stoichio:compound_id:compart
reactant	1.0:CPD-23708:c
product	1.0:CPD-23709:c
pathway	PWY-8191

reaction_id	RXN-21831
comment	Curation Sterols (Girard et al.)
reversible	false
linked_gene	
#reactant/product	#stoichio:compound_id:compart
reactant	1.0:CPD-23709:c
product	1.0:CPD-12850:c
pathway	PWY-8191

reaction_id	RXN11876
comment	Curation Sterols (Girard et al.)
reversible	false
linked_gene	EsuFWS1071_10-9
#reactant/product	#stoichio:compound_id:compart
reactant	1.0:CPD-12850:c
product	1.0:CPD-12852:c
pathway	PWY-8191

reaction_id	RXN11881
comment	Curation Sterols (Girard et al.)
reversible	false
linked_gene	EsuFWS528_9
#reactant/product	#stoichio:compound_id:compart
reactant	1.0:CPD-12852:c
product	1.0:CPD-12853:c
pathway	PWY-8191

reaction_id	RXN11878
comment	Curation Sterols (Girard et al.)
reversible	false
linked_gene	EsuFWS144_14-3
#reactant/product	#stoichio:compound_id:compart
reactant	1.0:CPD-12853:c
product	1.0:4-METHYL-824-CHOLESTADIENOL:c
pathway	PWY-8191

reaction_id	RXN-13709
comment	Curation Sterols (Girard et al.)
reversible	false
linked_gene	
#reactant/product	#stoichio:compound_id:compart
reactant	1.0:4-METHYL-824-CHOLESTADIENOL:c
product	1.0:CPD-4702:c
pathway	PWY-8239

reaction_id	RXN66-318
comment	Curation Sterols (Girard et al.)
reversible	false
linked_gene	EsuBft275_5
#reactant/product	#stoichio:compound_id:compart
reactant	1.0:CPD-4702:c
product	1.0:CPD-4581:c
pathway	PWY-8239

reaction_id	RXN66-319
comment	Curation Sterols (Girard et al.)
reversible	false
linked_gene	
#reactant/product	#stoichio:compound_id:compart
reactant	1.0:CPD-4581:c
product	1.0:ZYMOSTEROL:c
pathway	PWY-8239

reaction_id	RXN66-320
comment	Curation Sterols (Girard et al.)
reversible	true
linked_gene	EsuFWS87_17
#reactant/product	#stoichio:compound_id:compart
reactant	1.0:ZYMOSTEROL:c
product	1.0:5-ALPHA-CHOLESTA-724-DIEN-3-BETA-OL:c
pathway	PWY-8239

reaction_id	RXN-11887
comment	Curation Sterols (Girard et al.)
reversible	false
linked_gene	EsuFWS91_14
reactant	1.0:5-ALPHA-CHOLESTA-724-DIEN-3-BETA-OL:c
product	1.0:CPD-8646:c
pathway	PWY-8191

reaction_id	RXN-22206
comment	Gallo2020
reversible	false
linked_gene	EsuFWS91_14
reactant	1.0:CPD-24184:c
product	1.0:CPD-24185:c
pathway	PWY-8238

reaction_id	RXN-22198
comment	Gallo2020
reversible	false
linked_gene	EsuFWS166_11
reactant	1.0:CPD-24185:c
product	1.0:CPD-22095:c
pathway	PWY-8238

Table S11. List of reactions added in the genome-scale metabolic network of *S. japonica*

reaction_id	RXN-21829
comment	Curation Sterols (Girard et al.)
reversible	false
linked_gene	
#reactant/product	#stoichio:compound_id:compart
reactant	1.0:CYCLOARTENOL:c
product	1.0:CPD-23708:c
pathway	PWY-8191

reaction_id	RXN-21830
comment	Curation Sterols (Girard et al.)
reversible	false
linked_gene	
#reactant/product	#stoichio:compound_id:compart
reactant	1.0:CPD-23708:c
product	1.0:CPD-23709:c
pathway	PWY-8191

reaction_id	RXN-21831
comment	Curation Sterols (Girard et al.)
reversible	false
linked_gene	
#reactant/product	#stoichio:compound_id:compart
reactant	1.0:CPD-23709:c
product	1.0:CPD-12850:c
pathway	PWY-8191

reaction_id	RXN11876
comment	Curation Sterols (Girard et al.)
reversible	false
linked_gene	SJ09463
#reactant/product	#stoichio:compound_id:compart
reactant	1.0:CPD-12850:c
product	1.0:CPD-12852:c
pathway	PWY-8191

reaction_id	RXN11881
comment	Curation Sterols (Girard et al.)
reversible	false
linked_gene	SJ05072
#reactant/product	#stoichio:compound_id:compart
reactant	1.0:CPD-12852:c
product	1.0:CPD-12853:c
pathway	PWY-8191

reaction_id	RXN11878
comment	Curation Sterols (Girard et al.)
reversible	false
linked_gene	SJ12265
#reactant/product	#stoichio:compound_id:compart
reactant	1.0:CPD-12853:c
product	1.0:4-METHYL-824-CHOLESTADIENOL:c
pathway	PWY-8191

reaction_id	RXN-13709
comment	Curation Sterols (Girard et al.)
reversible	false
linked_gene	
#reactant/product	#stoichio:compound_id:compart
reactant	1.0:4-METHYL-824-CHOLESTADIENOL:c
product	1.0:CPD-4702:c
pathway	PWY-8239

reaction_id	RXN66-318
comment	Curation Sterols (Girard et al.)
reversible	false
linked_gene	
#reactant/product	#stoichio:compound_id:compart
reactant	1.0:CPD-4702:c
product	1.0:CPD-4581:c
pathway	PWY-8239

reaction_id	RXN66-319
comment	Curation Sterols (Girard et al.)
reversible	false
linked_gene	
#reactant/product	#stoichio:compound_id:compart
reactant	1.0:CPD-4581:c
product	1.0:ZYMOSTEROL:c
pathway	PWY-8239

reaction_id	RXN66-320
comment	Curation Sterols (Girard et al.)
reversible	true
linked_gene	
#reactant/product	#stoichio:compound_id:compart
reactant	1.0:ZYMOSTEROL:c
product	1.0:5-ALPHA-CHOLESTA-724-DIEN-3-BETA-OL:c
pathway	PWY-8239

reaction_id	RXN-11887
comment	Curation Sterols (Girard et al.)
reversible	false
linked_gene	SJ11625
reactant	1.0:5-ALPHA-CHOLESTA-724-DIEN-3-BETA-OL:c
product	1.0:CPD-8646:c
pathway	PWY-8191

reaction_id	RXN-22206
comment	Gallo2020
reversible	false
linked_gene	SJ11625
reactant	1.0:CPD-24184:c
product	1.0:CPD-24185:c
pathway	PWY-8238

reaction_id	RXN-22198
comment	Gallo2020
reversible	false
linked_gene	SJ02661
reactant	1.0:CPD-24185:c
product	1.0:CPD-22095:c
pathway	PWY-8238

Table S9. List of reactions added in the genome-scale metabolic network of *E. siliculosus*

reaction_id	RXN-21829
comment	Curation Sterols (Girard et al.)
reversible	false
linked_gene	
#reactant/product	#stoichio:compound_id:compart
reactant	1.0:CYCLOARTENOL:c
product	1.0:CPD-23708:c
pathway	PWY-8191

reaction_id	RXN-21830
comment	Curation Sterols (Girard et al.)
reversible	false
linked_gene	Esi0147_0062
#reactant/product	#stoichio:compound_id:compart
reactant	1.0:CPD-23708:c
product	1.0:CPD-23709:c
pathway	PWY-8191

reaction_id	RXN-21831
comment	Curation Sterols (Girard et al.)
reversible	false
linked_gene	
#reactant/product	#stoichio:compound_id:compart
reactant	1.0:CPD-23709:c
product	1.0:CPD-12850:c
pathway	PWY-8191

reaction_id	RXN11876
comment	Curation Sterols (Girard et al.)
reversible	false
linked_gene	Esi0169_0047
#reactant/product	#stoichio:compound_id:compart
reactant	1.0:CPD-12850:c
product	1.0:CPD-12852:c
pathway	PWY-8191

reaction_id	RXN11881
comment	Curation Sterols (Girard et al.)
reversible	false
linked_gene	Esi0006_0200
#reactant/product	#stoichio:compound_id:compart
reactant	1.0:CPD-12852:c
product	1.0:CPD-12853:c
pathway	PWY-8191

reaction_id	RXN11878
comment	Curation Sterols (Girard et al.)
reversible	false
linked_gene	Esi0073_0082
#reactant/product	#stoichio:compound_id:compart
reactant	1.0:CPD-12853:c
product	1.0:4-METHYL-824-CHOLESTADIENOL:c
pathway	PWY-8191

reaction_id	RXN-13709
comment	Curation Sterols (Girard et al.)
reversible	false
linked_gene
#reactant/product	#stoichio:compound_id:compart
reactant	1.0:4-METHYL-824-CHOLESTADIENOL:c
product	1.0:CPD-4702:c
pathway	PWY-8239

reaction_id	RXN66-318
comment	Curation Sterols (Girard et al.)
reversible	false
linked_gene	Esi0147_0062
#reactant/product	#stoichio:compound_id:compart
reactant	1.0:CPD-4702:c
product	1.0:CPD-4581:c
pathway	PWY-8239

reaction_id	RXN66-319
comment	Curation Sterols (Girard et al.)
reversible	false
linked_gene
#reactant/product	#stoichio:compound_id:compart
reactant	1.0:CPD-4581:c
product	1.0:ZYMOSTEROL:c
pathway	PWY-8239

reaction_id	RXN66-320
comment	Curation Sterols (Girard et al.)
reversible	true
linked_gene	Esi0155_0043
#reactant/product	#stoichio:compound_id:compart
reactant	1.0:ZYMOSTEROL:c
product	1.0:5-ALPHA-CHOLESTA-724-DIEN-3-BETA-OL:c
pathway	PWY-8239

reaction_id	RXN-11887
comment	Curation Sterols (Girard et al.)
reversible	false
linked_gene	Esi0003_0314
reactant	1.0:5-ALPHA-CHOLESTA-724-DIEN-3-BETA-OL:c
product	1.0:CPD-8646:c
pathway	PWY-8191

reaction_id	RXN-22206
comment	Gallo2020
reversible	false
linked_gene	Esi0003_0314
reactant	1.0:CPD-24184:c
product	1.0:CPD-24185:c
pathway	PWY-8238

reaction_id	RXN-22198
comment	Gallo2020
reversible	false
linked_gene	Esi0044_0110
reactant	1.0:CPD-24185:c
product	1.0:CPD-22095:c
pathway	PWY-8238

Table S10. List of reactions added in the genome-scale metabolic network of *E. subulatus*

reaction_id	RXN-21829
comment	Curation Sterols (Girard et al.)
reversible	false
linked_gene	EsuFWS8841_2 and EsuFWS13301_2
#reactant/product	#stoichio:compound_id:compart
reactant	1.0:CYCLOARTENOL:c
product	1.0:CPD-23708:c
pathway	PWY-8191

reaction_id	RXN-21830
comment	Curation Sterols (Girard et al.)
reversible	false
linked_gene	CACKRE030002836.1 and CAB1107767.1
#reactant/product	#stoichio:compound_id:compart
reactant	1.0:CPD-23708:c
product	1.0:CPD-23709:c
pathway	PWY-8191

reaction_id	RXN-21831
comment	Curation Sterols (Girard et al.)
reversible	false
linked_gene	
#reactant/product	#stoichio:compound_id:compart
reactant	1.0:CPD-23709:c
product	1.0:CPD-12850:c
pathway	PWY-8191

reaction_id	RXN11876
comment	Curation Sterols (Girard et al.)
reversible	false
linked_gene	EsuFWS1071_10-9
#reactant/product	#stoichio:compound_id:compart
reactant	1.0:CPD-12850:c
product	1.0:CPD-12852:c
pathway	PWY-8191

reaction_id	RXN11881
comment	Curation Sterols (Girard et al.)
reversible	false
linked_gene	EsuFWS528_9
#reactant/product	#stoichio:compound_id:compart
reactant	1.0:CPD-12852:c
product	1.0:CPD-12853:c
pathway	PWY-8191

reaction_id	RXN11878
comment	Curation Sterols (Girard et al.)
reversible	false
linked_gene	EsuFWS144_14-3
#reactant/product	#stoichio:compound_id:compart
reactant	1.0:CPD-12853:c
product	1.0:4-METHYL-824-CHOLESTADIENOL:c
pathway	PWY-8191

reaction_id RXN-13709
comment Curation Sterols (Girard et al.)
reversible false
linked_gene
#reactant/product #stoichio:compound_id:compart
reactant 1.0:4-METHYL-824-CHOLESTADIENOL:c
product 1.0:CPD-4702:c
pathway PWY-8239

reaction_id RXN66-318
comment Curation Sterols (Girard et al.)
reversible false
linked_gene EsuBft275_5
#reactant/product #stoichio:compound_id:compart
reactant 1.0:CPD-4702:c
product 1.0:CPD-4581:c
pathway PWY-8239

reaction_id RXN66-319
comment Curation Sterols (Girard et al.)
reversible false
linked_gene
#reactant/product #stoichio:compound_id:compart
reactant 1.0:CPD-4581:c
product 1.0:ZYMOSTEROL:c
pathway PWY-8239

reaction_id RXN66-320
comment Curation Sterols (Girard et al.)
reversible true
linked_gene EsuFWS87_17
#reactant/product #stoichio:compound_id:compart
reactant 1.0:ZYMOSTEROL:c
product 1.0:5-ALPHA-CHOLESTA-724-DIEN-3-BETA-OL:c
pathway PWY-8239

reaction_id RXN-11887
comment Curation Sterols (Girard et al.)
reversible false
linked_gene EsuFWS91_14
reactant 1.0:5-ALPHA-CHOLESTA-724-DIEN-3-BETA-OL:c
product 1.0:CPD-8646:c
pathway PWY-8191

reaction_id RXN-22206
comment Gallo2020
reversible false
linked_gene EsuFWS91_14
reactant 1.0:CPD-24184:c
product 1.0:CPD-24185:c
pathway PWY-8238

reaction_id RXN-22198
comment Gallo2020
reversible false
linked_gene EsuFWS166_11
reactant 1.0:CPD-24185:c
product 1.0:CPD-22095:c
pathway PWY-8238

Table S11. List of reactions added in the genome-scale metabolic network of *S. japonica*

reaction_id	RXN-21829
comment	Curation Sterols (Girard et al.)
reversible	false
linked_gene	
#reactant/product	#stoichio:compound_id:compart
reactant	1.0:CYCLOARTENOL:c
product	1.0:CPD-23708:c
pathway	PWY-8191

reaction_id	RXN-21830
comment	Curation Sterols (Girard et al.)
reversible	false
linked_gene	
#reactant/product	#stoichio:compound_id:compart
reactant	1.0:CPD-23708:c
product	1.0:CPD-23709:c
pathway	PWY-8191

reaction_id	RXN-21831
comment	Curation Sterols (Girard et al.)
reversible	false
linked_gene	
#reactant/product	#stoichio:compound_id:compart
reactant	1.0:CPD-23709:c
product	1.0:CPD-12850:c
pathway	PWY-8191

reaction_id	RXN11876
comment	Curation Sterols (Girard et al.)
reversible	false
linked_gene	SJ09463
#reactant/product	#stoichio:compound_id:compart
reactant	1.0:CPD-12850:c
product	1.0:CPD-12852:c
pathway	PWY-8191

reaction_id	RXN11881
comment	Curation Sterols (Girard et al.)
reversible	false
linked_gene	SJ05072
#reactant/product	#stoichio:compound_id:compart
reactant	1.0:CPD-12852:c
product	1.0:CPD-12853:c
pathway	PWY-8191

reaction_id	RXN11878
comment	Curation Sterols (Girard et al.)
reversible	false
linked_gene	SJ12265
#reactant/product	#stoichio:compound_id:compart
reactant	1.0:CPD-12853:c
product	1.0:4-METHYL-824-CHOLESTADIENOL:c
pathway	PWY-8191

reaction_id	RXN-13709
comment	Curation Sterols (Girard et al.)
reversible	false
linked_gene	
#reactant/product	#stoichio:compound_id:compart
reactant	1.0:4-METHYL-824-CHOLESTADIENOL:c
product	1.0:CPD-4702:c
pathway	PWY-8239

reaction_id	RXN66-318
comment	Curation Sterols (Girard et al.)
reversible	false
linked_gene	
#reactant/product	#stoichio:compound_id:compart
reactant	1.0:CPD-4702:c
product	1.0:CPD-4581:c
pathway	PWY-8239

reaction_id	RXN66-319
comment	Curation Sterols (Girard et al.)
reversible	false
linked_gene	
#reactant/product	#stoichio:compound_id:compart
reactant	1.0:CPD-4581:c
product	1.0:ZYMOSTEROL:c
pathway	PWY-8239

reaction_id	RXN66-320
comment	Curation Sterols (Girard et al.)
reversible	true
linked_gene	
#reactant/product	#stoichio:compound_id:compart
reactant	1.0:ZYMOSTEROL:c
product	1.0:5-ALPHA-CHOLESTA-724-DIEN-3-BETA-OL:c
pathway	PWY-8239

reaction_id	RXN-11887
comment	Curation Sterols (Girard et al.)
reversible	false
linked_gene	SJ11625
reactant	1.0:5-ALPHA-CHOLESTA-724-DIEN-3-BETA-OL:c
product	1.0:CPD-8646:c
pathway	PWY-8191

reaction_id	RXN-22206
comment	Gallo2020
reversible	false
linked_gene	SJ11625
reactant	1.0:CPD-24184:c
product	1.0:CPD-24185:c
pathway	PWY-8238

reaction_id	RXN-22198
comment	Gallo2020
reversible	false
linked_gene	SJ02661
reactant	1.0:CPD-24185:c
product	1.0:CPD-22095:c
pathway	PWY-8238
